# Cytoplasmic cyclin D1 regulates cell invasion and metastasis through the phosphorylation of paxillin

**DOI:** 10.1038/ncomms11581

**Published:** 2016-05-16

**Authors:** Noel P. Fusté, Rita Fernández-Hernández, Tània Cemeli, Cristina Mirantes, Neus Pedraza, Marta Rafel, Jordi Torres-Rosell, Neus Colomina, Francisco Ferrezuelo, Xavier Dolcet, Eloi Garí

**Affiliations:** 1 Cell Cycle Lab, Institut de Recerca Biomèdica de Lleida (IRBLleida), and Departament de Ciències Mèdiques Bàsiques; Facultat de Medicina; Universitat de Lleida, 25198 Lleida, Catalonia, Spain; 2 Oncopathology Lab, Institut de Recerca Biomèdica de Lleida (IRBLleida), and Departament de Ciències Mèdiques Bàsiques; Facultat de Medicina; Universitat de Lleida, 25198 Lleida, Catalonia, Spain

## Abstract

Cyclin D1 (Ccnd1) together with its binding partner Cdk4 act as a transcriptional regulator to control cell proliferation and migration, and abnormal Ccnd1·Cdk4 expression promotes tumour growth and metastasis. While different nuclear Ccnd1·Cdk4 targets participating in cell proliferation and tissue development have been identified, little is known about how Ccnd1·Cdk4 controls cell adherence and invasion. Here, we show that the focal adhesion component paxillin is a cytoplasmic substrate of Ccnd1·Cdk4. This complex phosphorylates a fraction of paxillin specifically associated to the cell membrane, and promotes Rac1 activation, thereby triggering membrane ruffling and cell invasion in both normal fibroblasts and tumour cells. Our results demonstrate that localization of Ccnd1·Cdk4 to the cytoplasm does not simply act to restrain cell proliferation, but constitutes a functionally relevant mechanism operating under normal and pathological conditions to control cell adhesion, migration and metastasis through activation of a Ccnd1·Cdk4-paxillin-Rac1 axis.

## Abstract (web summary)

Previous studies suggest that Cyclin D1 may regulate cell adhesion and migration but the mechanisms underlying such regulation and the relevance to cancer development are unknown. Here, Fusté *et al*. show that Cyclin D1/Cdk4 phosphorylates paxillin and thereby promotes cellular migration and metastasis.

## Graphical abstract

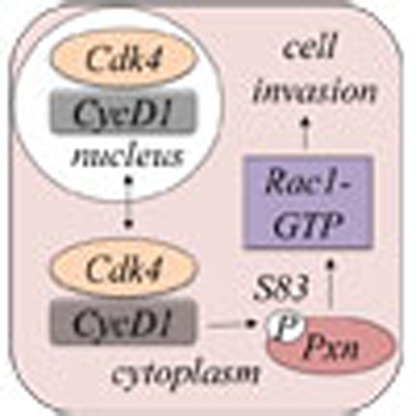

Cyclin D1 (Ccnd1) is a regulatory subunit of the cyclin-dependent kinases Cdk4/6, whose Ccnd1-dependent activity controls cell proliferation and development through its role as a transcriptional regulator^1^,^2^. Ccnd1 has been associated with tumour invasion and metastasis in clinical studies and in *in vivo* experiments^3^,^4^,^5^. This association seems related to the ability of Ccnd1 to regulate cell adhesion and migration, and not to the Ccnd1-dependent mechanisms that control cell proliferation^6^. *Ccnd1*^−/−^ mouse macrophages and fibroblasts show an increment in their capacity to adhere to the cell-matrix, and a reduction in cell motility^7^,^8^. These phenotypes have been attributed to the nuclear role of Ccnd1 as a transcriptional regulator of genes controlling cell adherence and migration^8^,^9^. However, the functional and physical interaction of Ccnd1 with cytoplasmic and membrane-associated proteins, such as filamin A, PACSIN2, RhoA and Ral GTPases indicate that this cyclin could play an active role in the cytoplasm regulating adherence and migration^10^,^11^,^12^,^13^,^14^.

The protein paxillin (Pxn) was identified as a tyrosine-kinase substrate and described as a structural and regulatory component of focal adhesions (FAs)^15^,^16^, the macromolecular assemblies through which the cytoskeleton connects to the extracellular matrix. The regulation of FA recycling is a key step in the control of cell adherence and motility^17^,^18^. Thus, Pxn-null fibroblasts display abnormal FA formation, delay in cell spreading and reduced migration, while mice deficient in Pxn show early embryonic lethality mainly due to the impairment of cell migration^19^. In recent years several works have highlighted the importance of Pxn not only as an organizer of FAs but also as a molecular scaffold coordinating different signalling pathways^20^. Pxn serves as a substrate for several serine-threonine kinases in response to adhesion stimuli and growth factors^21^,^22^,^23^,^24^, being regulated and playing functional roles at cellular locations distinct from FAs such as membrane ruffles^25^,^26^.

Ccnd1^−/−^ cells spread more rapidly, show an elevated number of adhesions sites centripetally distributed around the circumference of cells, and also exhibit augmented levels of tyrosine-phosphorylated Pxn^7^,^8^, suggesting the existence of alterations in the adhesion machinery. In this work, we show that the Ccnd1·Cdk4 complex phosphorylates a subpopulation of Pxn present in membrane ruffles but not in FAs, which is functionally relevant in the control of cell spreading and invasion in both normal fibroblasts and tumour cells. Although it is widely accepted that the accumulation of Ccnd1 in the cytoplasm operates only as a sequestration mechanism to prevent cell proliferation^27^,^28^, our results demonstrate that cytoplasmic Ccnd1 has an active role in the induction of cell migration and invasion. In addition, the existence of a Ccnd1·Cdk4-Pxn-Rac1 axis helps explain the invasive properties of tumours overexpressing Ccnd1.

## Results

### Pxn binds to and is an *in vitro* substrate of Ccnd1·Cdk4

**Figure 1:**
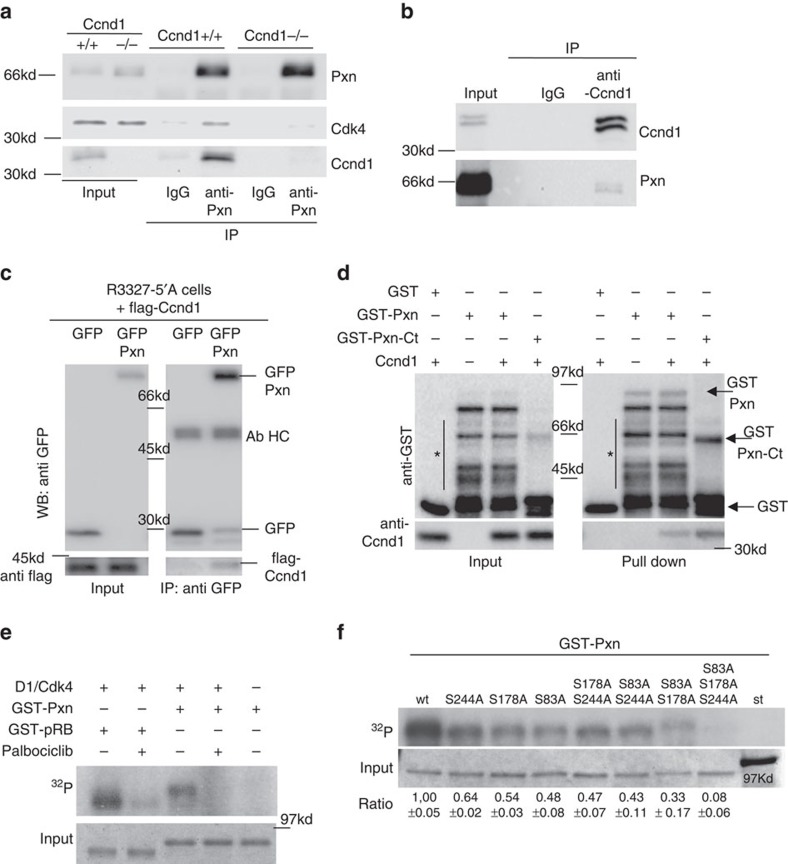
Pxn directly binds to and is an *in vitro* substrate of Ccnd1·Cdk4. (**a**) A rabbit polyclonal antibody (anti-Pxn) was used to IP endogenous Pxn from *Ccnd1*^−/−^ and *Ccnd1*^+/+^ fibroblasts. A rabbit polyclonal antibody against the Flag epitope (IgG) was used as a mock experiment. Input and IP samples were analyzed by western blot to detect Ccnd1, Cdk4 and Pxn. (**b**) IP with a rabbit polyclonal anti-Ccnd1, and anti-Flag (IgG) as a mock control in wild-type fibroblasts. (**c**) Rat prostate tumor cells R3327-5′A were co-transfected with GFP-tagged human Pxn, or an empty GFP vector, and Flag-tagged human Ccnd1. Cell lysates were immunoprecipitated with an anti-GFP monoclonal antibody, and immunoblotted with anti-GFP (top panel) or anti-Flag (bottom panel). Ab HC, antibody heavy chain. (**d**) The Ccnd1 protein produced by *in vitro* translation was incubated with GST or GST-Pxn fusion proteins (full length or C-terminal region containing the four LIM domains, aa337–591) purified from *E. coli*. Input and pull down samples were analyzed by western blot to detect Ccnd1 and GST. Asterisk indicates degradation bands. (**e**) Ccnd1·Cdk4 complexes (Sigma) were assayed for kinase activity against GST-Pxn (full length) and GST-Rb1 (aa379–928). The Cdk4/6 inhibitor Palbociclib was added at 2 μM. Coomassie blue staining was used to test equal loading (bottom panel). (**f**) Kinase assay as in E for different non-phosphorylatable mutants of Pxn. Band intensity was quantified with ImageJ under conditions of unsaturated signal exposure. The phosphorylation efficiency is shown as a ratio relative to wild type (mean±s.d.) of two independent experiments; st, size standard.

Depletion of Ccnd1 promotes cell attachment to the extracellular matrix, a process likely mediated through the stabilization of FAs^8^. Considering that FAs are central elements to the control of cell adherence and migration, we explored whether Ccnd1 could interact with FA components. In mouse fibroblasts, we found specific co-immunoprecipitation (co-IP) of both endogenous Ccnd1 and Cdk4 with Pxn (Fig. 1a), a key component of FAs^20^. In *Ccnd1*^−/−^ fibroblasts we were unable to co-IP Cdk4, even though the amount of immunoprecipitated Pxn was slightly higher than in wild-type cells. This likely indicates that Cdk4 must form a complex with Ccnd1 in order to interact with Pxn. IP of endogenous Ccnd1 also brought down Pxn in a specific way, albeit the amount of immunoprecipitated Pxn was very low compared with total Pxn in the whole cell extract (Fig. 1b). Our results are compatible with a significant amount of Ccnd1 interacting with a small fraction of total Pxn in the cell (see below). In addition, we have also observed the interaction between Pxn and Ccnd1 under heterologous conditions. We co-transfected both green fluorescent protein (GFP)-Pxn and Flag-Ccnd1 into rat prostate tumour cells (R3327-5′A) and performed an IP against GFP. The anti-GFP antibody was able to co-IP the flag-tagged Ccnd1 only when GFP was fused to Pxn, but not in control cells transfected with GFP alone (Fig. 1c). In order to test whether the interaction between Ccnd1 and Pxn is direct, we carried out *in vitro* GST-pull down assays. GST-fusions with full-length Pxn or only with the C-terminal domain of the protein purified from bacteria were mixed with Ccnd1 produced by *in vitro* translation. We recovered Ccnd1 bound to glutathione beads only when the fusion constructs were used, but not with GST alone (Fig. 1d). Overall, our results indicate that there is a specific and direct interaction between Pxn and Ccnd1·Cdk4 at endogenous levels in unperturbed cells.

Pxn is regulated by phosphorylation at different residues in response to a plethora of extracellular stimuli^20^. Because Pxn contains many putative Cdk-phosphorylation sites, we analyzed whether Pxn serves as a substrate for the Ccnd1·Cdk4 complex. Ccnd1·Cdk4 complexes purified from insect cells phosphorylated GST-Pxn obtained by heterologous expression in *E. coli* (Fig. 1e). Omission of the Ccnd1·Cdk4 complex or using the Cdk4/6 specific inhibitor Palbociclib prevented phosphorylation of GST-Pxn, confirming that the observed phosphorylation was due to the Ccnd1·Cdk4 complex included in the assay. To pinpoint the phosphorylated residues, we first studied the *in vitro* phosphorylation of deleted constructs, and next we created point mutations by site-directed mutagenesis. The analysis of these mutant versions of Pxn by *in vitro* phosphorylation allowed us to establish that Ccnd1·Cdk4 targets three different serines (S83, S178 and S244) in Pxn (Fig. 1f). In addition, we confirmed the phosphorylation at serine 83 by mass spectrometry (Supplementary Fig. 1A; Supplementary Tables 1 and 2). Failure to phosphorylate the mutated versions was not due to the lack of interaction, because we were still able to co-IP comparable amounts of hemagglutinin (HA)-tagged Ccnd1 with wild-type and mutant versions of GFP-tagged Pxn in co-transfected human HEK293T cells (see Supplementary Fig. 1B). Whereas the S244 residue is within a consensus sequence for the Cdk2 kinase, and it is phosphorylated by Cdk5 during oligodendrocyte differentiation^24^, phosphorylation of Pxn at serines 83 and 178 has been involved in the regulation of cell adhesion and migration. As Ccnd1 has a role in the control of cell adhesion and migration^7^,^8^, we have centred our study in the importance of phosphorylation at serines 83 and 178.

### Pxn phosphorylation by Ccnd1·Cdk4 in invasion and spreading

**Figure 2:**
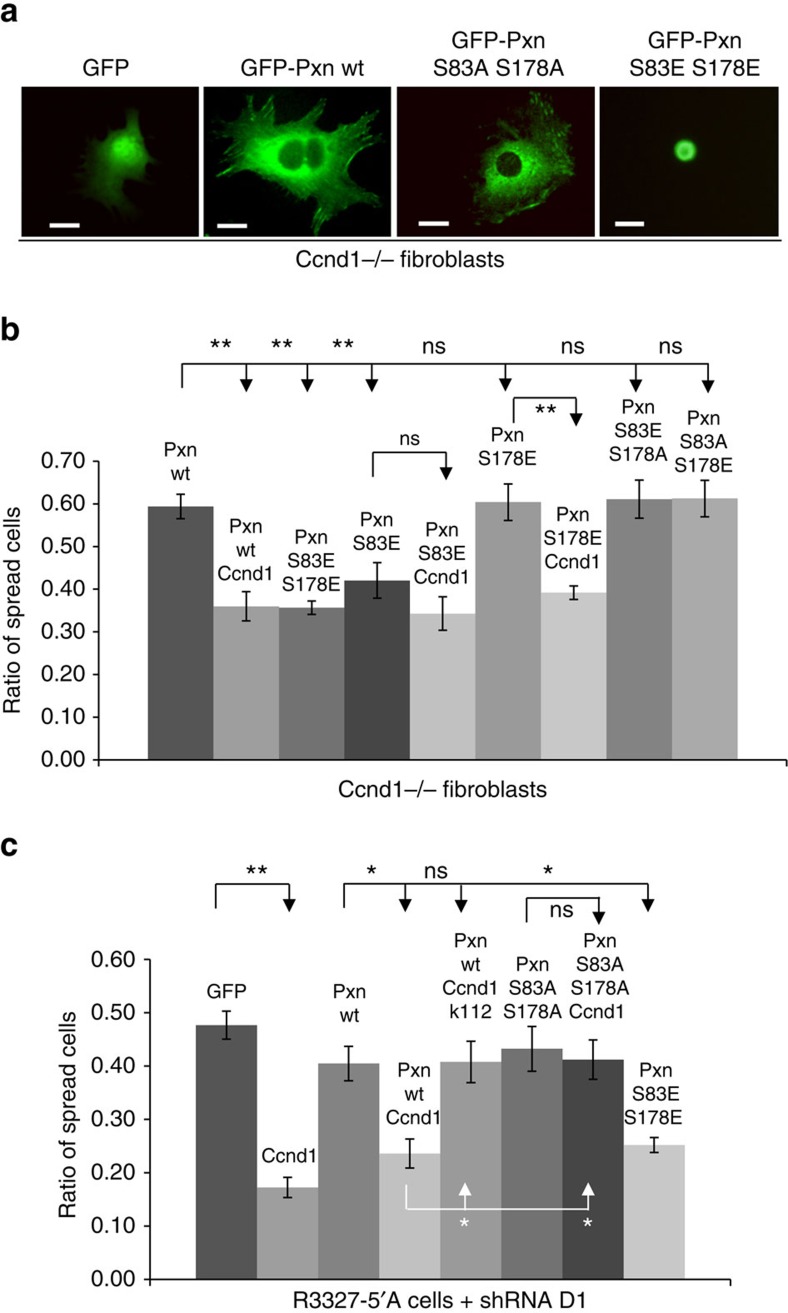
Pxn phosphorylation at serines 83 and 178 is required for Ccnd1-dependent delay in cell spreading. (**a**) Representative image of unspread and spread morphology in fibroblasts after transfection with various alleles of GFP-Pxn. (20 μm bar) (**b**,**c**) *Ccnd1*^−/−^ fibroblasts (**b**) or R3327-5′A cells expressing the shRNA·D1 (**c**) were co-transfected with an allele (wild type or mutant) of GFP-Pxn and either HA-Ccnd1 or an empty vector. Forty-eight hours after transfection, cells were trypsinized and seeded in serum-free medium in 35-mm well plates coated with 5 μg ml^−1^ fibronectin. Thirty minutes (fibroblasts) or one hour later (R3327-5′A cells), the proportion of spread green cells was determined (see Methods for details). Data (mean±s.e.m.) are from three or more independent experiments. Significance values were determined by one way ANOVA and Tukey-HSD post-test (*P<0.05, **P<0.01; ns, no significant).

**Figure 3:**
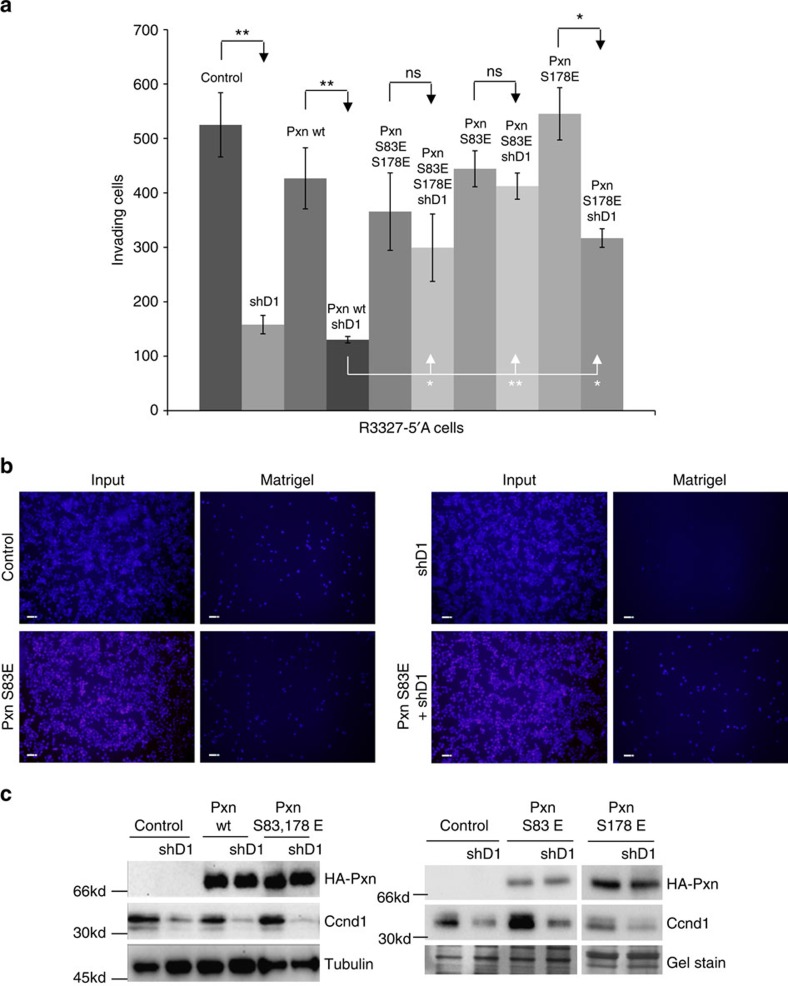
Ccnd1·Cdk4 regulates invasion through the phosphorylation of Pxn. (**a**) Prostate tumour cells (R3327-5′A) were infected with interference shRNA against Ccnd1 (shD1, Sigma) or with a scramble shRNA as a control. These cells were further infected with a wild type HA-Pxn, an S83, 178E HA-Pxn, an S83E HA-Pxn, an S178E HA-Pxn or with an empty vector, and 5 × 10^4^ co-infected cells were seeded in 24-well transwell filters previously coated with matrigel, and allowed to invade for 24 h (see Methods for more details). Relative values are expressed as mean±s.e.m. Data are from three independent experiments. Significance values were determined by one way ANOVA and Tukey-HSD post-test (*P<0.05, **P<0.01, ns no significant). (**b**) Representative images (50 μm bar) of the experiment in A. Cells were fixed and stained with Hoescht (input). Non-invading cells were removed using a cotton applicator (matrigel). (**c**) Immunoblots showing the expression of Pxn (rat anti-HA) and Ccnd1 (monoclonal antibody DCS6) in the co-infected cells. Tubulin or gel staining were used to test equal loading.

Ccnd1-deficient fibroblasts show the same diameter size than wild-type cells, but attach and spread more rapidly than these after seeded in fibronectin-coated plates^7^,^8^. Since Pxn is required for efficient and rapid spreading of fibroblasts in fibronectin^19^, we hypothesized that Ccnd1 could negatively regulate cell spreading through the phosphorylation of serines 83 and 178 in Pxn. In order to test this, we carried out functional assays with single and double phosphomimetic (serine to glutamic acid) and non-phosphorylatable (serine to alanine) Pxn mutants (see Figs 2 and 3). First, we transfected these mutants fused to GFP into *Ccnd1*^*−*/*−*^ fibroblasts, and green cells were evaluated for their spreading capacity (Fig. 2a,b). Under our assay conditions, expression of Ccnd1 produced a delay of spreading in otherwise *Ccnd1*^*−*/*−*^ fibroblasts. This effect was mimicked by the single phosphomimetic S83E allele (as well as the double mutant S83,178E) (Fig. 2b and Supplementary Fig. 2A). However, both the S83E S178A and the S83A S178E alleles with a non-phosphorylatable residue at position 178 or 83, respectively, did not delay spreading (see also Supplementary Fig. 2B). Hence, phosphorylation at both sites is required for Pxn-dependent control of cell spreading. Presumably, a kinase other than Ccnd1·Cdk4 must be responsible for the phosphorylation of serine 178 when Ccnd1^−/−^ cells are transfected with the single mutant S83E. By contrast, the single S178E mutant only had an effect on spreading when co-transfected with Ccnd1. This strongly suggests that the phosphorylation event of serine 83 that is relevant for the spreading effect depends on Ccnd1.

We also analyzed the importance of Ccnd1·Cdk4 in regulating the spreading of rat prostate tumour cells (R3327-5′A), which show an enhanced metastatic potential^29^. Downregulation of Ccnd1 by RNA interference significantly augmented the ability of R3327-5′A cells to spread in fibronectin-coated plates (Supplementary Fig. 3A,B). Expression of mutant alleles of Pxn in Ccnd1-deficient R3327-5′A cells produced similar results to those just described for fibroblasts (Fig. 2c), indicating that phosphorylation of Pxn by Ccnd1·Cdk4 is also important in the regulation of spreading in these cells. Expression of the Ccnd1K112E mutant, which cannot produce active Ccnd1·Cdk4 complexes, did not rescue the deficiency of Ccnd1 in R3327-5′A cells, corroborating that Ccnd1·Cdk4 kinase activity is required for cell-spreading control.

Ccnd1-deficient cells migrate and invade less than wild-type cells^8^,^10^. Since phosphorylation of Pxn at serines 83 and 178 is required for efficient migration^22^,^23^, we postulated that Ccnd1·Cdk4 may exert its positive effect on migration and invasion through Pxn phosphorylation. We analyzed the contribution of Ccnd1 to the ability of R3327-5′A cells to invade in matrigel-coated transwells. Downregulation of Ccnd1 by short hairpin RNA (shRNA) dramatically reduced the invasion capacity of these cells, but this was restored by the expression of single and double phosphomimetic mutants (Fig. 3). Contrary to what we observed in the spreading assays, both single phosphomimetic alleles (HA-Pxn S83E and HA-Pxn S178E) rescue the invasion capacity of Ccnd1-depleted cells (Fig. 3a), which suggests that Ccnd1 may regulate invasion through the phosphorylation of Pxn at serine 83 and 178.

### Reduced Pxn S83 phosphorylation in Ccnd1-deficient cells

**Figure 4:**
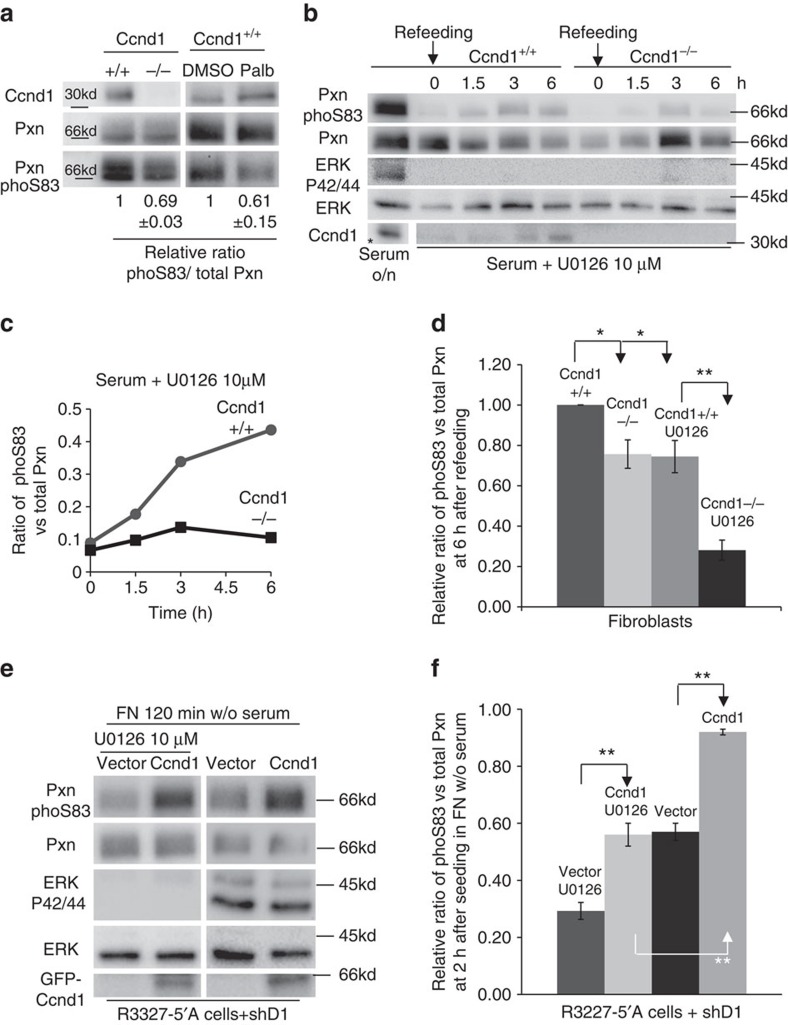
Ccnd1 knock-down leads to a reduction of phosphorylated Pxn at serine 83. (**a**) By immunoblot densitometry with the Image-Lab 4.0.1 software from BioRad, Pxn phosphorylated at serine 83 (phoS-83 Pxn) and total Pxn levels were determined in *Ccnd1*^−/−^ and *Ccnd1*^+/+^ fibroblasts, and in *Ccnd1*^+/+^ cells treated with 2 μM Palbociclib. Equal amounts of total protein per lane were loaded. Data are expressed as mean±s.e.m. (*n*=3). Significance was determined by a *t*-test. (**b**) Accumulation of phoS-83 and total Pxn in *Ccnd1*^−/−^ and *Ccnd1*^+/+^ fibroblasts after serum refeeding. At time zero cells were treated with 10 μM U0126, a specific inhibitor of the ERK pathway. Total ERK, P42/44 ERK, and Ccnd1 were examined in the same membranes. Wild-type fibroblasts cultured with serum were used as control. Asterisk indicates a lower exposure of the same membrane. (**c**) Quantification of the proportion of phosphorylated Pxn versus total Pxn from (**b**), by immunoblot densitometry as in (**a**). (**d**) The proportion of phosphorylated Pxn versus total Pxn at six hours after refeeding is plotted. Data are expressed as mean±s.e.m. (*n*=4). Significance was determined by one way ANOVA and Tukey-HSD post-test (*P<0.05, **P<0.01, ns not significant). (**e**) Accumulation of phoS-83 and total Pxn in R3327-5′A cells seeded in fibronectin. Cells, previously infected with shRNA against Ccnd1 (shD1), were transfected with vector or with Ccnd1, and were seeded in fibronectin-coated plates for 2 h in the absence of serum, and treated with DMSO or U0126. Total ERK, P42/44 ERK, and Ccnd1 were examined in the same membranes. (**f**) The proportion of phosphorylated Pxn versus total Pxn is plotted. Samples without ERK inhibitor and with inhibitor were loaded in different gels, but quantification was made relative to the same sample (expressing Ccnd1 and without U0126) loaded in all the gels. Data are expressed as mean±s.e.m. (*n*=4). Significance was determined by one way ANOVA and Tukey-HSD post-test (*P<0.05, **P<0.01; ns, not significant). Quantification by densitometry as in **a**.

Our functional assays indicate a prominent role of Ccnd1, via Pxn phosphorylation, in the regulation of both cell spreading and invasion. Particularly, our *in vitro* phosphorylation assays and functional results suggest that *in vivo* Ccnd1·Cdk4 phosphorylates Pxn at serine 83. Hence, we used a phospho-specific antibody to compare the levels of phosphorylated serine 83 in Pxn between wild-type and Ccnd1-deficient cells (Fig. 4a). Surprisingly, although we consistently observed a modest decrease (30–40%) in the phosphorylation of serine 83 in the absence of Ccnd1, there was still an important contribution to serine 83 phosphorylation that was independent of Ccnd1. This was true for Ccnd1^−/−^ fibroblasts, R3327-5′A tumour cells wherein Ccnd1 was knocked down with RNA interference (see also Supplementary Fig. 3C), or Ccnd1^+/+^ fibroblasts treated with 2 μM Palbociclib, a specific inhibitor of Ccnd1·Cdk4 (Fig. 4a).

ERK1/2 kinase phosphorylates Pxn at serine 83 in FAs^23^. To better highlight the contribution of Ccnd1 to serine 83 phosphorylation, we examined Pxn phosphorylation in the presence of the specific inhibitor of the extracellular-signal-regulated kinases (ERK) pathway activity U0126. Wild-type and Ccnd1^−/−^ fibroblasts were deprived from serum to bring to a minimum both Ccnd1 levels and ERK activity. After 24 h, cells were refed with serum containing 10 μM U0126, and at different time points samples were recovered for analysis by western blot (Fig. 4b). Quantification of phosphorylated Pxn at serine 83 versus total Pxn showed a much higher ratio of Pxn phosphorylation in Ccnd1^+/+^ cells than in Ccnd1^−/−^ cells, particularly after 6 h of incubation in serum when Ccnd1 had been clearly induced (Fig. 4c,d). Moreover, we assessed the role of Ccnd1 in promoting Pxn phosphorylation under conditions similar to the spreading assay. R3327-5′A rat cells previously infected with shRNA against Ccnd1 (shD1) were transfected with vector or with human Ccnd1 and were seeded in fibronectin-coated plates for 2 h. These cells were also incubated in the absence of serum and treated with DMSO or 10 μM U0126. Under these conditions, the expression of Ccnd1 promoted phosphorylation of Pxn at S83 even in the absence of ERK activity (Fig. 4e,f). Overall, these results are consistent with Ccnd1·Cdk4 phosphorylating a subpopulation of total Pxn, which may be functionally important in the regulation of cell spreading and invasion. Also, these results show that Ccnd1·Cdk4 and ERK can act independently on Pxn regulation.

### Cyclin D1 co-localizes with Pxn in the cell membrane

**Figure 5:**
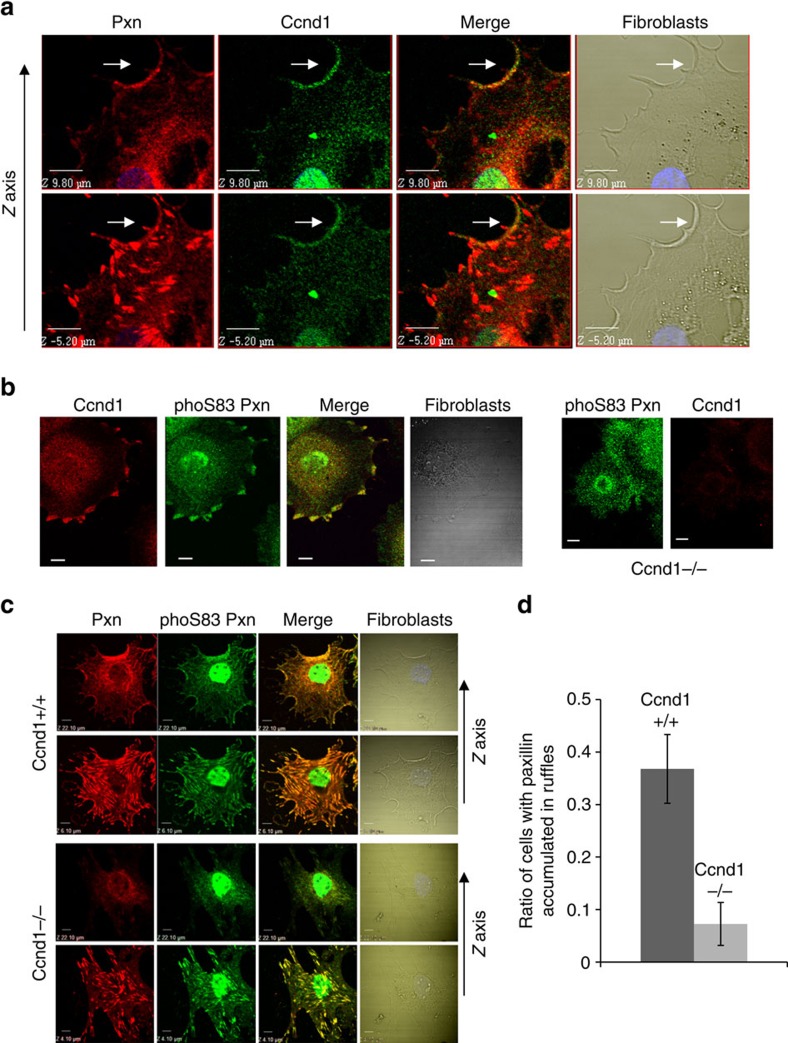
Ccnd1 co-localizes with Pxn in membrane ruffles of fibroblasts and tumour cells. (**a**) Fibroblasts were fixed in 4% paraformaldehyde and permeabilized with 0.2% Triton X-100. Images were acquired by confocal microscopy (10 μm bar). Nuclei were stained with Hoescht (blue). Anti-Ccnd1 (rabbit monoclonal clone EP12) and anti-Pxn (mouse monoclonal clone 349) antibodies were used. (**b**) Images of cultured *Ccnd1*^−/−^ and *Ccnd1*^+/+^ fibroblasts were processed as in A except for permeabilization (low conditions, 0.02% Triton X-100) (10 μm bar). Anti-Ccnd1 (mouse monoclonal clone 72-13 G) and anti-Pxn (S83) phospho-specific (rabbit polyclonal) antibodies were used. Note that the phospho-specific antibody against Pxn gives a nuclear signal that must be non-specific because total Pxn shows exclusion from the nucleus. (**c**) Images of cultured *Ccnd1*^−/−^ and *Ccnd1*^+/+^ fibroblasts were processed as in **a** (10 μm bar). Primary antibodies were the same as in **a** (anti-Pxn) and **b** (anti-Pxn S83). (**d**) Ratio of cells displaying Pxn accumulation in membrane ruffles, analyzed from the images in **c** (see also Supplementary Fig. S6 and Methods). The number of counted cells was *n*≥179. Bars indicate the confidence limits for a proportion (*α*=0.05).

Because our previous experiments had suggested that Ccnd1 may bind to and phosphorylate just a specific pool of cellular Pxn, we reasoned that Ccnd1–Pxn interaction may be constrained by their subcellular localization. This prompted us to study the localization of Ccnd1, Pxn and phospho-serine 83 Pxn by immunofluorescence and confocal microscopy. To this purpose, fibroblasts seeded on fibronectin-coated plates were incubated for 3 h and then fixed. As expected, we detected accumulation of Ccnd1 in the nucleus of most cells, but many also showed a diffuse cytoplasmic signal (Fig. 5a). Importantly, this diffuse signal was specific as judged by the almost complete absence of fluorescence in Ccnd1^−/−^ cells (Supplementary Fig. 4A). Interestingly, about 30% of cells showed co-localization of Ccnd1 with Pxn in the cell membrane. By contrast, we did not observe co-localization of Ccnd1 and Pxn at FAs. We also observed co-localization of Ccnd1 with S83-phosphorylated Pxn along the cell membrane of fibroblasts (Fig. 5b). The same result was obtained in tumour cells using an HA-tagged version of Ccnd1 (Supplementary Fig. 5).

Neither Ccnd1 nor Pxn seem to be homogeneously distributed throughout the cell membrane, but rather to specific regions of it. This is consistent with previous findings that associate both Ccnd1 and Pxn to membrane ruffles^10^,^13^,^26^. Rac1 is essential for the formation of membrane ruffles and can be used as a marker for these structures^30^. Hence, to determine if Ccnd1 localized to membrane ruffles, we studied the co-localization of Ccnd1 with Rac1. Again, about one-third of cells showed co-localization of Ccnd1 with Rac1 in the cell membrane, indicating that the interaction between Ccnd1 and Pxn very likely takes place at these ripples of the membrane (Supplementary Fig. 4B). Furthermore, membrane ruffles are regions with active membrane recycling and are thus enriched in the transferrin receptor protein (TFR)^31^. Accordingly, we examined co-localization of Ccnd1 with TFR in our cells. Once more, co-localization was only observed at specific regions in the cell membrane (Supplementary Fig. 4C), supporting our conclusion of a specific localization of Ccnd1 to membrane ruffles.

Finally, we checked whether the localization of Pxn was altered in Ccnd1^−/−^ fibroblasts. By immunofluorescence, both total and serine-83-phosphorylated Pxn were greatly reduced in the cell membrane in both immortalized and primary Ccnd1^−/−^ mouse embryonic fibroblasts (MEFs) (Fig. 5c,d; Supplementary Fig. 6A,B). By contrast, FAs retained a considerable amount of phosphorylated Pxn in these cells. This is consistent with the modest decrease in phosphorylation detected in western blots (see above, Fig. 4a). Therefore, it seems likely that Ccnd1·Cdk4 regulates only the fraction of Pxn located in membrane ruffles. Hence, we analyzed the localization of Ccnd1 and Pxn by cell fractionation. We obtained a soluble fraction containing cytosolic and nuclear soluble proteins, and a membrane-enriched fraction as indicated by protein markers for each fraction (Supplementary Fig. 7A). Both Ccnd1 and Pxn were found in the membrane fraction of fibroblasts (Supplementary Fig. 7A). To determine whether Ccnd1 and Cdk4 form active complexes in the membrane, we immunoprecipitated Ccnd1 from membrane fractions of R3327-5′A cells (which have higher endogenous levels of Ccnd1), and performed a kinase assay with those fractions using GST-Pxn as substrate. As shown in Supplementary Fig. 7, purified Ccnd1·Cdk4 complexes from the cell membrane are active, at least as measured by our *in vitro* assays.

### Rac1 activity is downregulated in Ccnd1-deficient cells

**Figure 6:**
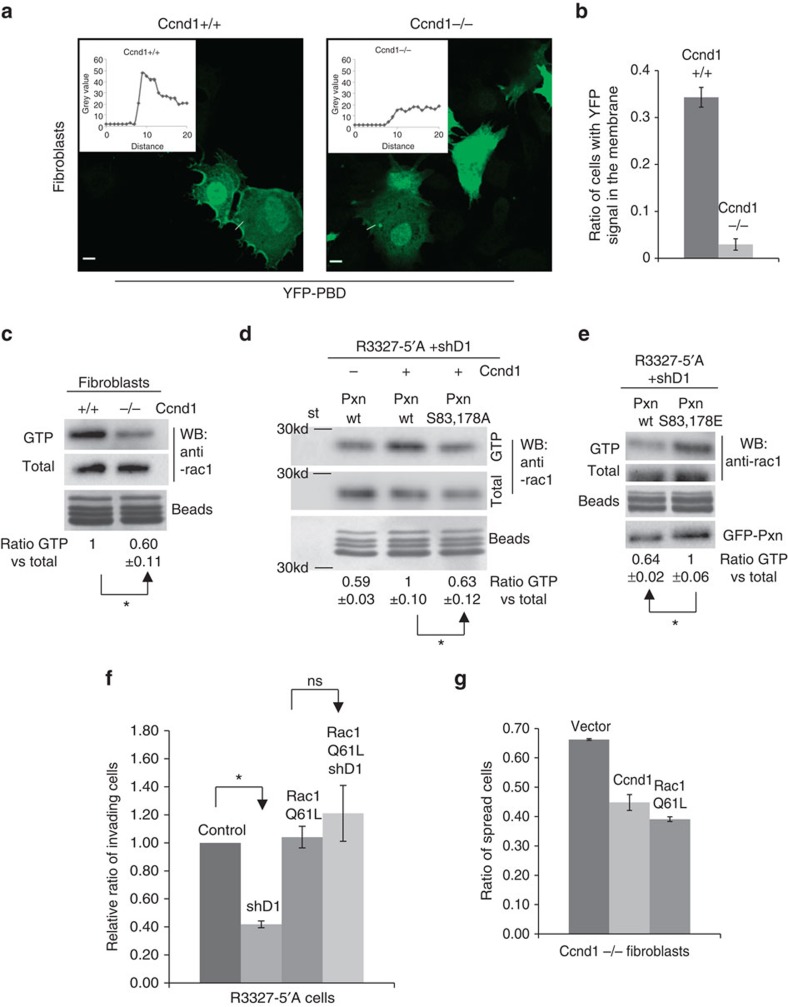
Ccnd1 knock-down cells show reduced levels of Rac1 activation in the membrane. (**a**) *Ccnd1*^−/−^ and *Ccnd1*^+/+^ fibroblasts transfected with YFP-PBD were seeded in fibronectin-plates for 1 h and then fixed 10 min on ice in 2% PFA to avoid YFP-signal loss (10 μm bar). We measured YFP signal with Image J. Two representative measures are shown. (**b**) Ratio of cells displaying YFP-signal accumulation in membrane calculated from two independent experiments (mean±s.d.). Cells counted: *n*≥ 200. (**c**) Quantification of Rac1 activity in *Ccnd1*^−/−^ and *Ccnd1*^+/+^ fibroblasts by Rac1-GTP pull-down assay. Values show densitometric analysis of relative activity of Rac1 normalized for whole cell lysates (mean±s.e.m.; *n*=4). Significance was determined by a *t*-test (*P<0.05). (**d**) R3327-5′A cells previously infected with shRNA against Ccnd1 (shD1, Sigma) and transfected with Pxn or with Pxn S83,178A, and human Ccnd1 were used in Rac1 pull-down assays. Relative values are expressed as mean±s.e.m. (*n*=4). Significance was determined by a *t*-test (*P<0.05). (**e**) The same cells as in **d** were transfected with wild type Pxn or with the phosphomimetic Pxn S83,178E mutant, and were used in Rac1 pull-down assays. Relative values are expressed as mean±s.e.m. (*n*=3). Significance was determined by a *t*-test (*P<0.05). (**f**) R3327-5′A cells were infected first with shD1 or with a scramble shRNA as a control, then they were further infected with the hyperactive allele Rac1Q61L or with an empty vector, and cells were seeded in 24-well transwell filters previously coated with matrigel, and allowed to invade for 24 h. Relative values are expressed as mean±s.e.m. (*n*=3). Significance was determined by one way ANOVA and Tukey-HSD post-test (*P<0.05; ns, no significant). (**g**) *Ccnd1*^−/−^ fibroblasts were transfected with Rac1Q61L or HA-Ccnd1 or an empty vector. Forty-eight hours after transfection, cells were seeded in serum-free medium in fibronectin-coated plates. Thirty minutes later the proportion of spread green cells was determined and plotted. Data are from two independent experiments and expressed as mean±s.d.

To varying degrees, Ccnd1^−/−^ cells show more spread morphology than the corresponding wild type and also exhibit augmented number of FAs^7^,^8^, with higher levels of tyrosine-phosphorylated Pxn. In contrast, we have observed less accumulation of Pxn in the membranes of Ccnd1^−/−^ cells in this work (Fig. 5c). This decrease could be in fact a consequence of morphological changes in these cells, such as a reduction of membrane ruffles. Rac1 GTPase is the major inductor of membrane ruffling and is required for cell migration and invasion^26^. Pxn induces migration and Rac1 activation through several mechanisms that promote the recruitment of Rac1-associated guanine nucleotide exchange factor (GEF) activity to the leading edge of the cells^20^. Since Ccnd1·Cdk4 regulates Pxn phosphorylation and cell invasion, we hypothesized that Ccnd1·Cdk4 could alter Rac1 activity via Pxn phosphorylation. Then, a reduction on Ccnd1 levels could lead to a reduction of membrane ruffles, and a concomitant decline in cell invasion. Therefore, we examined both the localization and activity of Rac1 in Ccnd1^−/−^ cells. We transfected wild type and Ccnd1^−/−^ fibroblasts with a yellow fluorescent protein-Pak1 binding domain (YFP-PBD) construct that acts as a fluorescent biosensor of Rac1 activity^32^. Forty-eight hours after transfection, we seeded the cells in fibronectin for 1 h, then fixed them, and processed for immunofluorescence (IF). Although some transfected cells showed strong diffuse YFP or nuclear signal, a clear accumulation of YFP-PBD signal in the membranes of wild-type fibroblasts (Fig. 6a) was generally observed. This signal co-localizes with Rac1 and Ccnd1 (Supplementary Fig. 8). Importantly, few Ccnd1^−/−^ cells showed YFP signal in membranes (Fig. 6a,b). A similar result was obtained by analyzing total Rac1 in immortalized and primary MEFs (Supplementary Fig. 9). In addition, we detected a drop of activated Rac1 (by 40%) in Ccnd1^−/−^ fibroblasts, even though the total levels of Rac1 remained unchanged in these cells (Fig. 6c). These results strongly suggest that Rac1 is active in membranes of normal fibroblasts during spreading but not in Ccnd1^−/−^ cells. In R3327-5′A rat tumour cells that had Ccnd1 downregulated by RNA interference, activated Rac1 levels depended on Ccnd1 because transfecting these cells with human Ccnd1 restored the wild-type levels of activated Rac1. However, co-transfecting human Ccnd1 with a non-phosphorylatable allele of Pxn (S83,178A) had no effect on the levels of activated Rac1 (Fig. 6d and Supplementary Fig. 9E). In addition, the expression of a phosphomimetic allele of Pxn (S83,178E) restored Rac1 activation in Ccnd1 knock-down cells (Fig. 6e). Hence, these results indicate that activation of Rac1 GTPase by Ccnd1 is mediated by Pxn phosphorylation. Also, the decrease in Rac1 activation might be responsible for the morphological alterations in Ccnd1^−/−^ cells.

If Ccnd1 exerts its effects on migration through a pathway that leads to Rac1 regulation, then hyperactivation of Rac1 should rescue the invasion phenotype of Ccnd1-deficient cells. Consequently, we tested whether expression of a hyperactivated allele of Rac1 (Rac1Q61L) was able to recover the invasion potential of Ccnd1-compromised R3327-5′A cells. We found that indeed this is the case (Fig. 6f). Also, the expression of Rac1Q61L produced a delay in the spreading of Ccnd1^−/−^ fibroblasts comparable to those transfected with Ccnd1 (Fig. 6g). Taken together these results suggest that Ccnd1 regulates cell migration in a cascade of events that lead to Rac1 activation through Pxn phosphorylation.

### Phospho-Pxn restores metastases by Ccnd1-deficient cells

**Figure 7:**
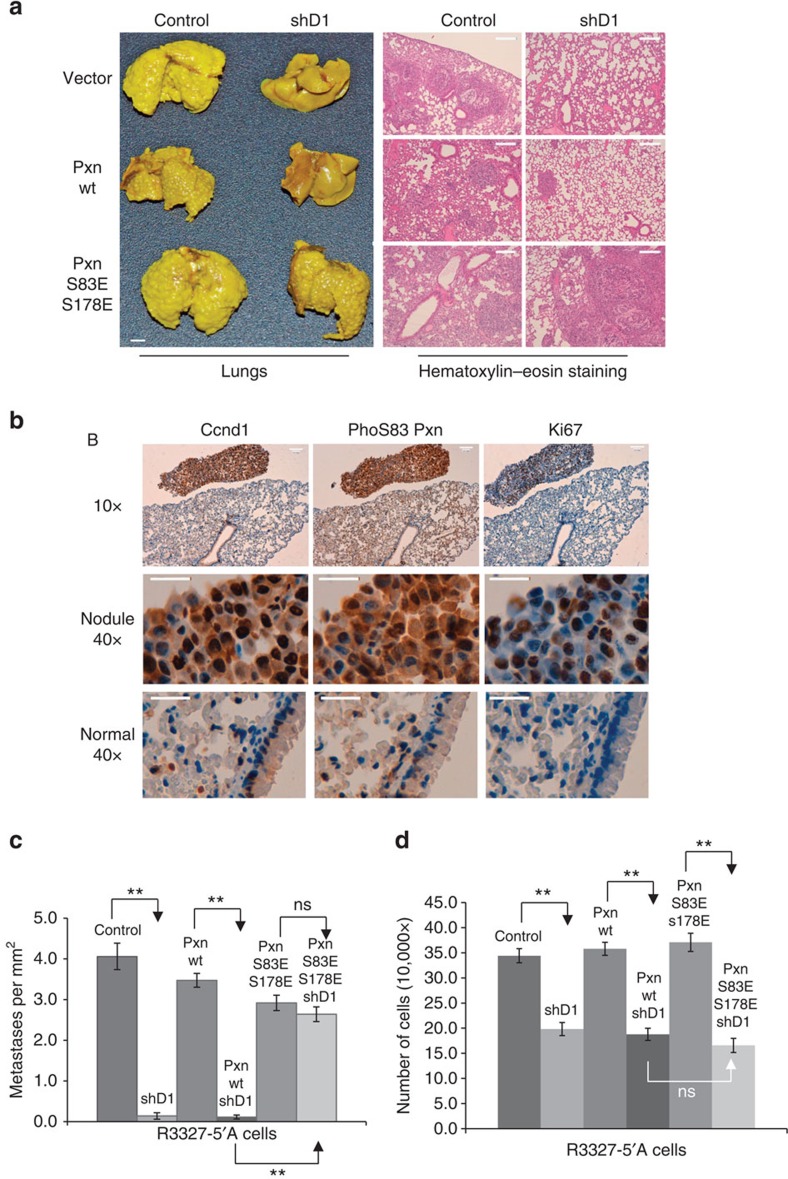
Phosphomimetic Pxn rescues the low metastatic potential of Ccnd1-deficient cells. (**a**) For metastasis assays, 5 × 10^5^ co-infected R3327-5′A cells as in Fig. 3 were inoculated in nude mice (four animals per condition) by retroorbital intravenous injection, and the animals were euthanized fifteen days later. Lungs were recovered, fixed in Bouin's solution (3 mm bar), and a sample was included in paraffin for hematoxylin–eosin staining (200 μm bar). (**b**) A piece of biopsy was fixed with formaldehyde at 4%, included in paraffin and processed by immunohistochemistry (IHC) to detect Ccnd1, phosphorylated Pxn and Ki67 (25 μm bar). (**c**) Metastatic capacity was evaluated as number of metastases per mm^2^, and expressed as mean±s.e.m. (*n*=4) with significance values determined by one way ANOVA and Tukey-HSD post-test (**P<0.01; ns no significant). (**d**) For proliferation assays, 2 × 10^4^ co-infected cells were seeded and grown in DMEM 10% serum at 37 °C, 5% CO_2_. After three days, cell number was determined and plotted. The experiment was repeated five times and data are expressed as mean±s.e.m. with significance values determined by one way ANOVA and Tukey-HSD post-test (**P<0.01).

Ccnd1 is a marker of poor prognosis and has been associated with metastasis in clinical studies^3^. Ccnd1-deficient cells consistently show a reduced metastatic potential^4^,^5^. Because our results point to Pxn as a mediator of the effects due to Ccnd1 on cell adherence and migration, we wanted to test whether a phosphomimetic version of Pxn was able to rescue the low metastatic potential of Ccnd1-deficient cells. To this end, we performed a metastasis assay *in vivo* by bloodstream injection of R3327-5′A cells in 12-weeks-old nude mice. Animals were euthanized 2 weeks after injection, and their lungs examined both macro- and microscopically (Fig. 7a). Tumour masses show high levels of nuclear and cytoplasmic Ccnd1 and phosphorylated Pxn at serine 83 (Fig. 7b). Downregulation of Ccnd1 by RNA interference drastically reduced R3327-5′A-dependent metastases. Strikingly, the presence of a phosphomimetic S83,178E Pxn allele rescued the metastatic potential of these cells (Fig. 7a,c). This effect was not due to changes in the proliferative potential of the cells. Ccnd1 is important to maintain a high-proliferation rate in transformed cell lines and, as expected, downregulating Ccnd1 reduced the proliferative capacity of R3327-5′A cells to a half (Fig. 7d). Yet, a similar reduction in proliferation was observed in cells expressing the phosphomimetic allele of Pxn. Therefore, the rescue of the metastatic potential cannot be attributed to changes in proliferation. We then propose that phosphorylation of Pxn by Ccnd1·Cdk4 is a new mechanism whereby Ccnd1 promotes metastasis.

## Discussion

The best studied role of the Ccnd1·Cdk4 complex is as a regulator of transcription in the nucleus. However, some studies have also proposed a cytoplasmic function for the complex^10^,^12^,^13^. The accumulation of Ccnd1·Cdk4 outside the nucleus was initially described as a mechanism to arrest cell proliferation. For instance, oncogenic Ras induces re-localization of Ccnd1·Cdk4 in the cytoplasm and promotes proliferation arrest in primary human keratinocytes, probably as a mechanism of defence against Ras-driven neoplasia^27^. Another example is the regulation of proliferation by tight junctions through the sequestration of Ccnd1·Cdk4 in the membrane of MDCK-epithelial cells^28^. Here, we show that the localization of Ccnd1·Cdk4 in the membrane of fibroblasts and tumour cells has an active role in the induction of cell migration and invasion through the phosphorylation of Pxn. Our findings do not exclude the re-localization of Ccnd1·Cdk4 as a mechanism of proliferation control, but indicate the existence of cytoplasmic substrates of Ccnd1·Cdk4; moreover, we show that Ccnd1·Cdk4 is involved in the regulation of cell-matrix adhesion and cell migration through the phosphorylation of a subpopulation of cytoplasmic Pxn molecules. Ccnd1 binds to and co-localizes with Pxn in the cell membrane, its removal or inhibition leads to decreased levels of Pxn phosphorylation, and it phosphorylates Pxn *in vitro*. In particular, Ccnd1-dependent phosphorylation of Pxn at serine 83 *in vivo* is required and irreplaceable in the regulation of cell spreading and invasion. By contrast, the effect on cell spreading of serine-178 phosphorylation, although required, does not rely entirely on Ccnd1. In this respect, c-Jun N-terminal kinase (JNK) kinase has been shown to promote cell spreading and migration in epithelial cells through the phosphorylation of Pxn at serine 178 (refs 22, 33). Also, we have observed that Ccnd1·Cdk4 *in vitro* phosphorylates Pxn at S244. Although phosphorylation at this site has not been associated with cell adhesion and migration, we cannot rule out the possibility that the phosphorylation at S244 by Ccnd1̇Cdk4 could play a role *in vivo* in those processes.

Phosphorylation of Pxn at serine 83 by Erk promotes cell spreading and migration^23^. However, Erk and Ccnd1·Cdk4 have a discordant effect on cell spreading modulation. Hepatocyte growth factor (HGF)-induced Erk activation promotes cell spreading in epithelial cells,^23^ whereas knockdown of Ccnd1·Cdk4 enhances cell spreading in mouse fibroblasts. This discrepancy could be related to the different localization of S83-phosphorylated Pxn. We have observed S83-phospho-Pxn both at the cell membrane and in FAs, but we have detected co-localization with Ccnd1 only at the membrane. By contrast, localization of Erk to FAs has been described^23^. Note that Ccnd1 binds to the C-terminal region (LIM domains) of Pxn (Fig. 1d), and that LIM domains are required for efficient targeting of Pxn to FAs^34^. Then, both interactions could be mutually exclusive. Conceivably, Pxn phosphorylation by Erk at FAs may lead to more efficient cell spreading while Pxn phosphorylation by Ccnd1 at the cell membrane may lead to an opposite effect. This is not a far-out possibility. For instance, Y31/118-phosphorylated Pxn is present at different locations promoting different effects on cell adhesion^26^. The tyrosine kinases FAK and Brk1 phosphorylate Pxn at FAs and lamellipodia, respectively, and both promote cell invasion. However, phosphorylation of Pxn by FAK is important for FA turnover and cell adhesion whereas phosphorylation of Pxn by Brk1 does not alter cell adhesion^26^.

Rac1 function is essential for membrane ruffling and protrusive activity of cells^35^. Similar to Ccnd1^−/−^ cells, Rac1^−/−^ fibroblasts show a more spread morphology than wild-type cells and are impaired in migration^36^. In this work, we propose that Ccnd1·Cdk4 induces Rac1 activation through the phosphorylation of Pxn at serine 83 in the cell membrane. We show that Ccnd1-deficient fibroblasts not only have a reduction in Pxn phosphorylated at serine 83 but also in Rac1-GTP levels, and exhibit a decrease in membrane ruffling after seeding in fibronectin. In addition, the expression of a hyperactive allele of Rac1 rescues the invasion and spreading phenotypes observed in Ccnd1-deficient cells to the same degree as a phosphomimetic allele of Pxn. Importantly, Ccnd1·Cdk4 only induces Rac1 activation in the presence of a wild-type allele of Pxn but not in the presence of an S83,178A non-phosphorylatable Pxn mutant. This result raises the question as to how S83-phosphorylated Pxn activates Rac1. In FAs, phosphorylation of Pxn at serine 83 by Erk enhances the interaction of Pxn with FAK, which consequently promotes the phosphorylation of Pxn at tyrosines 31 and 118 (ref. 23). In turn, phosphorylation of Pxn at these tyrosines leads to the recruitment of a GEF factor (Dock180) that induces Rac1 activity and cell migration^20^. This mechanism cannot be applied in our case because Ccnd1^−/−^ fibroblasts show an increase in the amount of Pxn phosphorylated at tyrosine 118 (ref. 8), while we have observed lower levels of activated Rac1 in Ccnd1^−/−^ cells. Then there must be alternative pathways involving other GEFs recruited by Pxn (such as β-PIX and Vav2) that could mediate Ccnd1-dependent activation of Rac1 (refs 20, 37).

At first, cyclins, Cdks and Cdk-inhibitors were exclusively viewed as nuclear proteins involved in cell cycle transitions. However, emerging data demonstrate that these cell cycle regulators also operate in the cytoplasm regulating cell functions independently of their cell cycle role. One of these functions is cell migration, which requires a tight coordination between Rho and Rac1 activities^35^. Rho activity has to be reduced, whereas Rac1 activity has to be increased in the leading edge of the cell. The Cip/Kip inhibitor p27 induces migration through the binding and inhibition of RhoA activity in the cytoplasm^38^. Also, the INK4 Cdk-inhibitor p16 promotes cell migration in hepatocellular carcinoma cells through the activation of Rac1, although it has also been described that p16 plays a negative role on migration in other tumours^39^. Finally, the Ccnd1·Cdk4 complex promotes cell migration through different cytoplasmic mechanisms. Pestell and collaborators have previously shown that Ccnd1 regulates cell migration by transcriptional repression of RhoA effectors^8^, and by modulating p27 levels, which inhibits RhoA signalling in the cytoplasm^9^. In this work we show that Ccnd1·Cdk4 promotes cell migration through Rac1 activation in the cytoplasm. Therefore, Ccnd1·Cdk4 seems to favour cell migration through the regulation of two types of Rho small GTPases, RhoA and Rac1, in an opposite way. Altogether, it seems that cell cycle regulators show a dual functionality regulating proliferation in the nucleus and migration in the cytoplasm. Perhaps, this feature is relevant in cell fate decision during development. Ccnd1 accumulates in the cytoplasm of post-mitotic neurons^40^ and during skin differentiation^41^ where it could act through Pxn to regulate migratory events throughout development.

The entrance of a malignant tumour in a metastatic process is often intractable and is a major concern in cancer therapeutics. Metastatic cells modify their ability to adhere to the extracellular matrix and acquire proficient motility. Ccnd1-dependent activity is frequently increased during tumour growth and metastasis^3^,^4^,^5^,^42^. Its importance in these processes has been attributed not only to its role in the control of cell proliferation but also to the regulation of cell-matrix adhesion and cell migration^6^. Here, we have demonstrated that the inhibitory effect on the metastatic potential of cells upon downregulation of Ccnd1 can be reverted by the expression of a phosphomimetic version of Pxn. This agrees with the reported requirement of phospho-Pxn for the induction of metastases in different tumour models^43^,^44^,^45^,^46^. Our data place Pxn downstream of Ccnd1, as a phosphorylation target, in a pathway regulating cell spreading, invasion and metastasis. This implies that accumulation of Ccnd1 in the cytoplasm would exert a key activating role on the metastatic potential, a correlation that has been observed in prostate tumours^47^ and in cancer cell lines capable of undergoing metastasis in *in vivo* models^48^. At present, the cyclin D·Cdk4,6 complexes are considered relevant targets for cancer therapy and at least three different specific inhibitors of Cdk4/6 are being used in different clinical trials^49^. For instance, Palbociclib has already been approved for the treatment of oestrogen receptor-positive, HER2-negative metastatic breast cancer. Our data reinforce the importance of these inhibitors in cancer therapy suggesting that the inactivation of Cyclin D·Cdk4,6 not only should produce tumour regression, as expected from its role in cell proliferation, but should restrict tumour spreading and metastasis as well.

## Methods

### Cell culture

*CCND1*^*−*/*−*^ and *CCND1*^*+/+*^ fibroblasts and R3327-5′ rat tumour cells were kindly provided by P. Sicinski and M. Hendrix respectively (growth conditions, morphology and chromosome number were authenticated following provider's instructions). HEK293T cells were obtained from the American Type Culture Collection. Primary MEFs were isolated from E14.5 wild type or *CCND1*^*−/−*^ embryos. All the cells were routinely tested for mycoplasma by PCR and maintained mycoplasma free. Our cell lines are not listed in ICLAC database. Cells were maintained at 37 °C in a 5% CO_2_ incubator, and grown in Dulbecco's modified Eagle's medium (DMEM) supplemented with 10% FBS, 100 μg ml^−1^ penicillin/streptomycin and 2 mM glutamine. Transient transfection of vectors was performed with Lipofectamine 2000 (Invitrogen) according to manufacturer's instructions. For lentivirus production, HEK293T cells were transfected with lentiviral expression vectors, envelope plasmid pVSV.G, and packaging plasmid pHR'82ΔR at a ratio 2:1:1. Stable knockdown of Ccnd1 was carried out by lentiviral infection of R3327-5′A cells and selection in 5 μg ml^−1^ of puromycin.

### Expression vectors

Both human *CCND1* wild type and the inactive allele *K112E* were fused to three copies of the FLAG epitope under the CMV promoter in pcDNA3. Also, the human CCND1 was used to obtain an N-terminal 3 × HA fusion under the UBI promoter in a lentiviral vector derived from pDSL (Invitrogen). Mouse *Pxn* (IMAGE ID 5309957 clone from Source BioScience) was used to obtain an N-terminal GST fusion in pGEX-KG (Clontech) or an N-terminal 3 × HA fusion under the UBI promoter in pDSL-derivative vector. GST-Pxn contains full length Pxn, while GST-Pxn-Ct contains only the four LIM domains of the protein (aa337-591). Both Pxn wild type and the S178E allele inserted into the mammalian GFP-tagged expression vector pEGFP-C1 (BD Biosciences) were kindly provided by J. Yamauchi^50^. Standard PCR-mediated site-directed mutagenesis was used to obtain the non-phosphorylatable and phosphomimetic mutants of Pxn using the following primers: GCCCCGCAGCGAGTCACCTCCAG and GCCCATCTCTCCCTGGTTCACAGT for S244A; GCGCCACTGCCCGTGTACAGCTC and CGGAGGCTGCTGGTGAGCGT for S83A; GCGCCCCTTTATGGCATCCCAGA and CAGGGCTCCAGGCAAGGGGGG for S178A; CCGGTGTACAGCTCCAGTGCTAA and TAGTGGCTCCGGAGGCTGCTGGTGAGCGT for S83E; GAGCCCCTTTATGGCATCCCAGA and GAGGGCTCCAGGCAAGGGG for S178E. The single, double and triple mutants were done in pGEX-KG (GST-Paxillin) and then transferred to pcDNA3 and pDSL vectors. The GST-pRb1 (aa379–928) fusion was a gift from N. Agell. The pCEFL-AU5-Rac1(Q61L) plasmid was obtained from P. Crespo via X. Bustelo. For RNA interference the CCND1 MISSION shRNA TRCN0000026883 cloned in a pLKO.1-puro was obtained from Sigma. The YFP-PBD vector was obtained from J. Swanson via Addgene.

### Immunofluorescence

Briefly, cells were quickly washed in PBS and fixed in 4% paraformaldehyde for 15 min at room temperature. Fixed MEFs and R3327-5′A cells were permeabilized with 0.2% Triton-X-100 for 3 min at room temperature, and blocked with 3% BSA. In Fig. 5b low permeabilization conditions were used (0.02% Triton-X-100). Primary antibodies at a working dilution 1:200 were combined with adequate Alexa488 and/or Alexa594-labelled secondary antibodies (Molecular Probes) in PBS with 0.3% BSA. Nuclei were stained with Hoechst (Sigma). Images were acquired using 40X and 60X objectives in an Olympus FV1000 confocal system. Primary antibodies used in IF were the following: anti-Ccnd1 (rabbit monoclonal clone EP12, Dako #M3642/ mouse monoclonal clone 72-13 G, Santa Cruz #sc450), anti-Pxn (S83) phospho-specific (polyclonal #PP1341, ECM Biosciences), anti-TFR (monoclonal H68.4, Invitrogen #13–6800), anti-Pxn (monoclonal 349, BD Biosciences #610051), anti-Rac1 (mouse monoclonal, clone 102, BD #610650) and anti-HA (rat monoclonal 3F10, Roche #11867431001, 1:1000). For detection of YFP-PBD we have used two different anti-GFPs antibodies (mouse monoclonal 3E6, Invitrogen #A11120, 1:200; rabbit Alexa Fluor 488 conjugate, Invitrogen #A21311, 1:400). The quantification of cells with Pxn and Rac1 accumulation in ruffles was carried out with Image J software (see Supplementary Fig. 6).

### Immunohistochemistry

Lung tissue samples were fixed with paraformaldehyde. Blocks were sectioned at a thickness of 3 μm and dried for 1 h at 65 °C, before being dewaxed in xylene and rehydrated through a graded ethanol series, then washed with PBS. Antigen retrieval was performed by heat treatment in a pressure cooker for 2 min in EDTA (pH 8.9). Before staining the sections, endogenous peroxidase was blocked. The antibodies used were anti-Ccnd1 (rabbit monoclonal clone EP12, Dako #M3642, 1:400), anti-Pxn S83 phospho-specific (polyclonal #PP1341, ECM Biosciences, 1:100), and anti-K67 (rabbit monoclonal clone SP6, Abcam #ab16667, 1:250). After incubation, the reaction was visualized with the EnVision Detection Kit (Dako), using diaminobenzidine chromogen as a substrate.

### Cell fractionation and immunoprecipitation

Protein fractionation was performed with the Subcellular Protein Fractionation kit for cultured cells (Thermo Scientific-Pierce; 78840). Soluble fraction corresponds to a mixture of cytosolic and nuclear soluble fractions described in the supplier's instructions.

IP of endogenous Ccnd1 was carried out in MEFs with a rabbit polyclonal antibody (#06–137, Upstate, 5 μg). IP of endogenous Pxn was done in Ccnd1^−/−^ and Ccnd1^+/+^ immortalized MEFs with an anti-Pxn rabbit polyclonal antibody (H-114, Santa Cruz #sc-5574, 2 μg). Briefly, cleared cell extracts in lysis buffer (10 mM Tris–HCl, pH 7.5, 50 mM NaCl, 1% Triton X-100, 3 mM MgCl_2_, 300 mM Sucrose, and protease and phosphatase inhibitors) were immunoprecipitated with Protein A linked to magnetic beads (Dynabeads, Invitrogen). An anti-FLAG (#F7425, Sigma) was used as a mock control. The same lysis buffer was used for IP of GFP-tagged Pxn in transfected R3327-5′A cells. Cleared extracts were immunoprecipitated with a mixture of anti-GFP monoclonal antibodies (Roche).

### Immunoblotting

For immunoblot, protein samples were resolved by SDS-PAGE, transferred to PVDF membranes (Millipore), and incubated with primary antibodies anti-Ccnd1 (monoclonal DCS-6, BD Pharmigen #556470, 1:500), anti-Cdk4 (polyclonal C-22, #sc-260, 1:250), anti-Pxn (monoclonal 349, BD transduction lab. #610051, 1:1000), anti-GFP (monoclonals 7.1 and 13.1, Roche #11814460001, 1:2000), anti-Flag (monoclonal M2, Sigma #F3165, 1:2000), anti-Pxn (ser-83) phospho-specific (polyclonal #PP1341, ECM Biosciences, 1:500), anti-HA (rat monoclonal 3F10, Roche #11867431001, 1:2000), anti-PCNA (monoclonal PC10, Abcam #ab29, 1:1000), anti-TFR (monoclonal H68.4, Invitrogen #13–6800, 1:1,000), anti-GAPDH-Peroxidase (monoclonal clone 71.1, Sigma #G9295, 1:40000), anti-ERK1/2 (monoclonal MK12, Merck #05–1152, 1:2000), anti-phospho-ERK1/2 (Thr202/Tyr204, polyclonal, Cell Signalling #9101, 1:800), anti-GST (goat polyclonal, Amersham #27–4577, 1:2000) and anti-tubulin (monoclonal B-5-1-2 Sigma #T5168, 1:10000). Appropriate peroxidase-linked secondary antibodies (GE Healthcare UK Ltd) were detected using the chemiluminescent HRP substrate Immobilon Western (Millipore). Chemiluminescence was recorded with a ChemiDoc-MP imaging system (BioRad). Uncropped scans of the most important western blots are supplied in the Supplementary Figs 10–13.

### GST pull-down assay

Flag tagged Ccnd1 was transcribed with T7 RNA polymerase (New England Biolabs) and translated in Rabbit Reticulocyte Lysate System (Promega). For the pull-down assay, 400 ng of GST or GST-Pxn purified from *E. coli* were immobilized on Glutathione-Sepharose 4B beads and incubated with Flag-Ccnd1 in binding buffer (20 mM HEPES-KOH, pH 7.5, 150 mM KCl, 5 mM MgCl_2_, 0.5 mM EDTA, 0.1% NP-40, 1 mM DTT, 1 mM PMSF, 10% glycerol, protease and phosphatase inhibitors) for 30 min at room temperature. After four washes with the same buffer, the samples were analyzed by SDS-PAGE.

### Kinase assay

Kinase reaction and purification of GST-Pxn substrates from *E. coli* were done as previously described^12^. Briefly, 0.2 μg substrate (either GST-Pxn or GST-pRb1) was mixed with 1.5 μl of active Ccnd1-Cdk4 complex purified from baculovirus (Sigma C0620), 10 μM ATP, 7 μCi of γ-32P-ATP (PerkinElmer, 3000 Ci/mmol), and either DMSO or 2 μM of the Cdk4/6-inhibitor Palbociclib (Selleckchem, S1116) in 20 μl of kinase buffer (50 mM Tris–HCl pH 7.5, 10 mM MgCl_2_, 0.5 mM DTT, 1 mM EGTA and 2.5 mM β-glycerophosphate). This mixture was incubated for 20 min at 30 °C, then boiled in 2 × Laemmli buffer, and separated by electrophoresis. Phosphorylated proteins were visualized by autoradiography of the dried slab gels. For substrate purification, *E. coli* cells transformed with GST fusions were grown to saturation overnight, diluted 1:10 in LB broth, and incubated at 37 °C for 2 h. GST-fusion proteins were induced by addition of IPTG for 4 h at 30 °C, after which cells were recovered by centrifugation at 4 °C and lysed on ice by sonication in 1 ml of lysis buffer (50 mM HEPES, pH7.5, 150 mM NaCl, 1 mM EDTA, 1 mM DTT, 10% glycerol, 0.5% Triton X-100). Cleared lysates were mixed with Glutathione-Sepharose 4B (GE, Healthcare) and incubated for 2 h at 4 °C. Beads were washed three times with lysis buffer and twice with kinase buffer, and the GST-fusion proteins were released by incubation with 2 mM reduced glutathione (Sigma). The concentration and purity of substrates were estimated by comparison to protein standards stained with Coomassie blue.

### In-gel digestion

Full length Pxn fused to GST was used in an *in vitro* kinase assay with Ccnd1-Cdk4 in the presence of ATP or in the absence of ATP as a control. Samples were subsequently subjected to SDS-PAGE. The gels were stained with Coomassie Brilliant Blue G-250 colloidal (EZBlue Gel Staining Reagent, Sigma). After washing with water, protein bands of interest were prepared and submitted to Fundació Institut d'Investigació Biomèdica de Bellvitge (IDIBELL) Proteomics Service (Barcelona) for analysis of the Chymotryptic peptide molecular masses by liquid chromatography-mass spectrometry. Gel slices were manually cut and proteomic service suggested for each band-assay to recover three slices (low 1, middle 2, and high 3 mobility) as phosphorylation could alter band mobility.

Briefly, gel bands were washed with water, ammonium bicarbonate (50 mM) and 50% acetonitrile. Next, samples were reduced by incubation with dithiothreitol (10 mM) at 60 °C for 45 min and alkylated with iodoacetamide (50 mM) for 30 min, in the dark. Finally, proteins were digested with chymotrypsin (5 ng μl^−1^) at 25 °C overnight (Trypsin gold, Promega). Digestion was stopped by addition of 5% formic acid and peptides extracted twice with 70% acetonitrile and 5% formic acid (10 min sonication). Peptide extracts were evaporated to dryness, resuspended with 2% acetonitrile 0.1% formic acid and analyzed by nano-HPLC-MSMS.

### LC-MSMS and database searching

Peptides were analyzed using an Easy-nanoLCII (Proxeon, Denmark) coupled to an Amazon ETD Ion trap (Bruker Daltonics). Peptides were first trapped on an Easy column TM C18 (2 cm, 5 μm, 100 μm ID, Thermo Scientific), and then separated using an analytical C18 nanocapillary column (75 μM ID, 15 cm, AcclaimPepMap 100 Thermo Scientific). The chromatography gradient was achieved by increasing percentage of buffer B from 0–35% at a flow rate of 300 nl min^−1^ over 40 min (A: 0.1% formic acid, B: 0.1% formic acid, 100% acetonitrile). Eluted peptides were then introduced into Amazon ETD Ion trap by electrospray ionization in the Captive-Spray ion Source (Bruker Daltonics) with an applied voltage of 1,500 V and N_2_ as drying gas. Peptide masses (400–1,400 *m*/*z*) were analyzed at full scan at enhanced resolution (range 50–3,000 *m*/*z* and speed 8.100 *m*/*z* sec) and 10 most intense peptides were selected and fragmented in a three-dimensional ion trap using both collision induced dissociation and electron transfer dissociation fragmentation, using as a collision gas hellium and methane, respectively. Data was generated with Data Analyst 4.1 software (Bruker Daltonics). MS and MS/MS data were analyzed with Protein Scape 3.1.2 software (Bruker Daltonics) using Mascot 2.4.0 (Matrix Science) as the search engine and SwissProt database (2015-03, 547,964 sequences; 195,174,196 residues). The specific parameters for protein sequence database searching included taxonomy *Mus musculus* (16,711 sequences), serine, threonine and tyrosine phosphorylation and methionine oxidation as variable modifications and cysteine carbamylation as a fixed modification. Other search parameters were: chymotrypsin digestion with two missed cleavages, charge states +1, +2 +3 for precursor ion, mass error of 0.6 Da for precursor ion and for fragment ions. For the identification a significance threshold peptide decoy (Mascot) of *P*<0.05 was set and only peptides with a minimum Mascot score of 25 and proteins with a minimum Mascot score of 35 were considered. Spectra from phosphorylated peptides were also manually examined.

### Rac1 pull-down assay

The assays were performed by using PAK1 PBD agarose-beads (Cell Biolabs, STA-411) according to the manufacturer's instructions. Cell lysates were obtained from one 100 mm plate from fibroblasts or tumour cells. The lysis buffer used was 50 mM Tris pH 7.5, 200 mM NaCl, 2.5 mM MgCl2, 2.5 mM DTT, 1% Triton and protease and phosphatase inhibitors. Lysates (0.6 ml) were incubated with 10 μg of PAK1 beads during 30 min at 4 °C and, after several washes, agarose beads were resuspended in 2 × Laemmli buffer. Samples were separated by SDS-PAGE, transferred to PVDF membranes, and immunoblotted.

### Cell spreading assay

Fibroblasts or R3327-5′A cells were co-transfected with GFP-Pxn wild type or any of the mutated alleles together with HA-Ccnd1 or an empty vector. Petri dishes were coated overnight at 4 °C with a 5 μg ml^−1^ solution of fibronectin (Invitrogen) in PBS. Forty-eight hours after transfection, cells were trypsinized and seeded in serum-free medium in fibronectin-coated 35-mm well plates. Fibroblasts were incubated for thirty minutes and R3327-5′A cells for 1 h, and then cells were fixed with 2% paraformaldehyde for 10 min on ice, imaged, and cells that had spread counted (*n*≥150 total green cells evaluated in each independent experiment). Round and bright cells were considered to be unspread.

### Cell invasion assay

We performed cell invasion assays with R3327-5′A cells as previously described^51^. Briefly, 6.5-mm filters of 8.0 pore size (Transwell, Corning) were coated with Matrigel (reduced-factors, BD Biosciences) in the upper side. Then, infected cells (5 × 10^4^) were seeded in the bottom side of the filter for four hours to allow their attachment. Afterwards, filters were loaded with DMEM 10% serum and incubated in 24-well plates containing serum-free medium for 24 h. Under these conditions, some cells migrate from the bottom to the upper side of the filter invading the Matrigel. The remaining cells at the bottom of the filter were removed, and Matrigel-embedded cells were fixed and stained with Hoescht. We have counted all cells in the filter extension with the Image J software.

### Metastasis assay

The procedure performed in this study followed the National Institutes of Health Guidelines for the Care and Use of Laboratory Animals, and is according to the Article 17.2 of the Law 5/1995 and the Article 33.a of Decree 214/1997 of 30 July, which regulate the use of animals for experimental and other scientific purposes (Catalan Government), and was certified by the Ethics Committee on Animal Experimentation from the University of Lleida (CEEA 03-03/13). Immunodeficient female SCID hr/hr mice (12-week-old; 20–25 g) were maintained in specific pathogen free conditions, and were inoculated with 5 × 10^5^ co-infected cells by retroorbital intravenous injection. Animals were euthanized fifteen days afterwards. Lungs were recovered and fixed in Bouin's solution; a sample was included in paraffin for hematoxylin–eosin staining.

### Statistical analyses

The investigators were blinded when assessing the outcome for experiments of spreading (Fig. 2b), invasion (Fig. 3a) and immuno-localization (Fig. 6b). Comparisons among groups were made by one way ANOVA and Tukey-HSD post-test or with two-tailed *t*-test allowing unequal variance (*P<0.05, **P<0.01, ns no significant). Throughout the paper, error bars indicate s.e.m. except for figures where we only have two independent experiments, such as Figs 1f and 6b,f (mean±s.d.). In Fig. 5d and Supplementary Figs 6B, 9B,D error bars refer to the confidence limits for a proportion.

For animal studies we have used the Ene 3.0: Program to calculate sample size. This software was developed by the Department of Applied Statistics of Autonomous University of Barcelona and is distributed by GlaxoSmithKline. No specific method of randomization was used but the group allocation was done randomly. The program suggested a minimum of 18 mice distributed among six groups (at least three animals per group) to get a 0.8 power to detect differences in the null hypothesis *H*_0_ (the mean of six groups they are equal) by one factor ANOVA test for independent samples, considering the significance level is 0.05. We have used 24 animals (four animals per group).

## Additional information

**How to cite this article:** Fusté, N. P. *et al*. Cytoplasmic cyclin D1 regulates cell invasion and metastasis through the phosphorylation of paxillin. *Nat. Commun.* 7:11581 doi: 10.1038/ncomms11581 (2016).

## Supplementary Material

Supplementary Information Supplementary Figures 1-13 and Supplementary Tables 1-2
