# Supplementary material for: Cytoplasmic cyclin D1 regulates cell invasion and metastasis through the phosphorylation of paxillin
Source: Nat Commun. 2016 May 16;7:11581. doi: 10.1038/ncomms11581 (PMC4873647; doi:10.1038/ncomms11581)
Supplement: Supplementary Information — Supplementary Figures 1-13 and Supplementary Tables 1-2 [file ncomms11581-s1.pdf]

SUPPLEMENTARY FIGURE 1

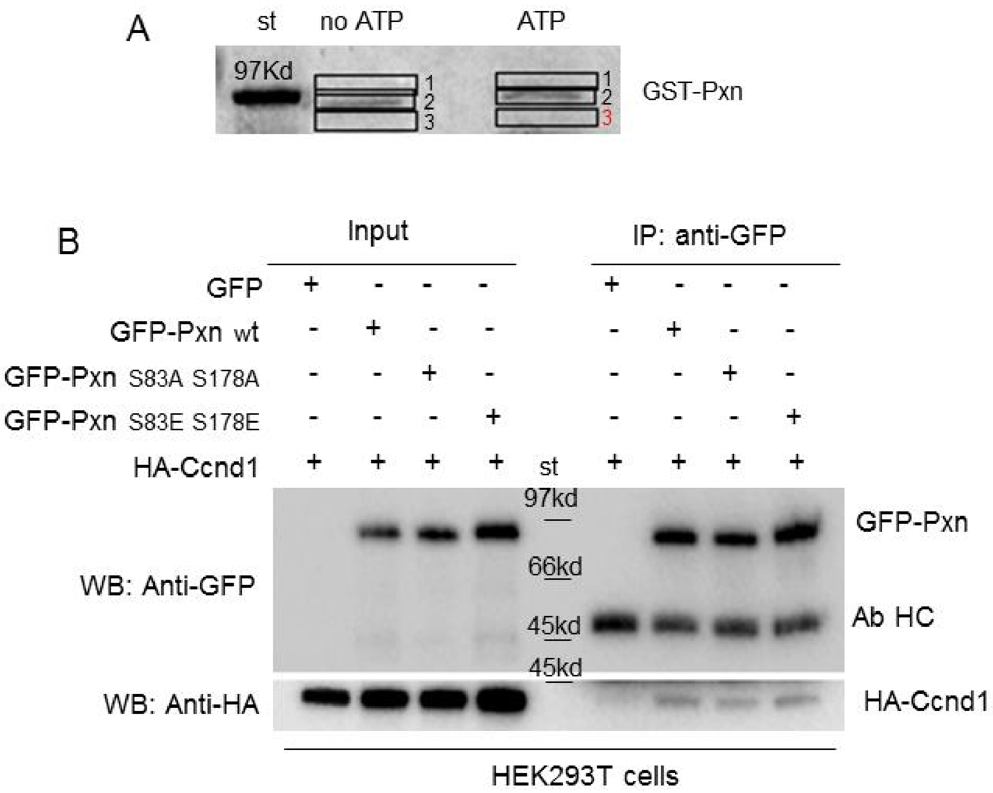

Supplementary Figure 1. Paxillin directly binds to and is an in vitro substrate of Cyclin D1·Cdk4. (A) Full length Pxn fused to GST was used in an in vitro kinase assay with Ccnd1-Cdk4 in the presence of ATP or in the absence of ATP (no ATP) as a control. Samples were subsequently subjected to SDS-PAGE. The gels were stained with Coomassie Brilliant Blue G-250 colloidal (EZBlue™ Gel Staining Reagent, Sigma). After washing with water, gel slices were manually cut and for each assay three slices were recovered (low 1, middle 2, and high 3 mobility bands) as phosphorylation could alter band mobility. Bands were sent for mass spectrometry analysis. **(B)** Cyclin D1 binds to wild-type and mutant versions of GFP-paxillin. HEK293T cells were co-transfected with various alleles of GFP-tagged human paxillin or empty GFP vector and HA-tagged human Ccnd1. Cell lysates were subject to IP with an anti-GFP monoclonal antibody and proteins detected by immunoblot with anti-GFP (top panel) or anti-HA (bottom panel) antibodies.

SUPPLEMENTARY FIGURE 2

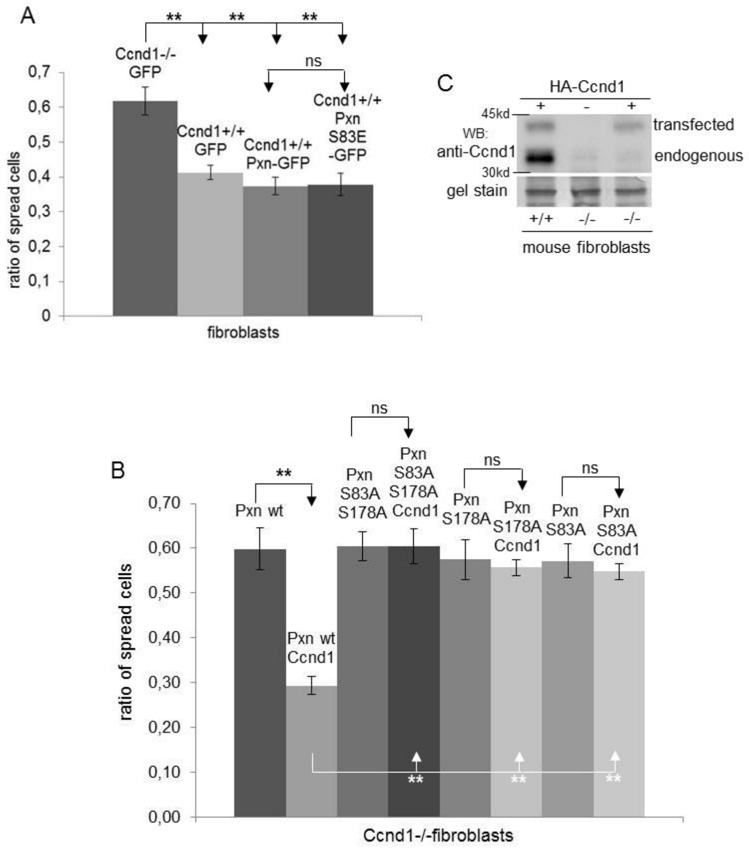

Supplementary Figure 2. Paxillin phosphorylation at serines 83 and 178 is required for cyclin D1-dependent delay in cell spreading. (A) Ccnd1$^{+/+}$ fibroblasts were co-transfected with GFP or GFP-Pxn wt or GFP-Pxn S83E. Also, Ccnd1$^{-/-}$ fibroblasts were transfected with GFP. Forty-eight hours after transfection, cells were trypsinized and seeded in serum-free medium in 35-mm well plates coated with 5 μg/ml fibronectin. Thirty minutes later the proportion of spread green cells was determined. For each condition, data from at least three independent experiments are plotted as mean ± SEM. Significance values are determined by one way ANOVA and Tukey-HSD post-test (**p<0.01). (B) Ccnd1$^{-/-}$ fibroblasts were co-transfected with different wild type or mutant versions of GFP-Pxn and with HA-Ccnd1, and processed as in A (mean ± SEM; n≥3). (C) Immunoblot to estimate the level of HA-Ccnd1 transfected in B. Considering a transfection efficiency of 20% and that transfected band is around the 20% of the endogenous (measured with Image-label program BioRad), we could assume that there is no a significant overexpression.

SUPPLEMENTARY FIGURE 3

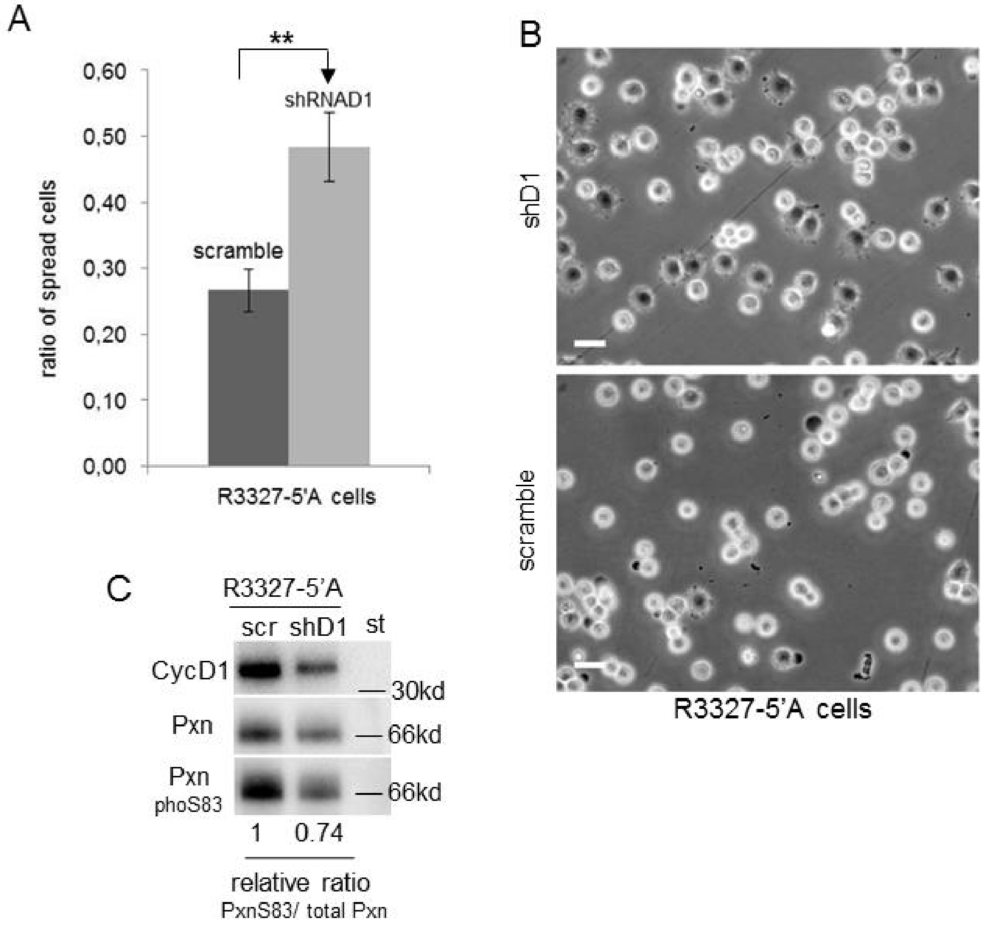

Supplementary Figure 3. Cyclin D1-dependent delay of spreading in tumor cells. (A) Rat prostate tumor R3327-5'A cells were infected with an interference shRNA against Ccnd1 (shD1, Sigma) or with a scramble shRNA as a control. Cells expressing the shD1 were selected with puromycin and used for a spreading assay. They were trypsinized and seeded in serum-free medium in 35-mm well plates coated with 5 μg/ml fibronectin. One hour later the proportion of spread green cells was determined. Data from three independent experiments are plotted as mean ± SEM. Significance values are determined by a t-test. (B) Representative image of R3327-5'A cells from one of the experiments described in A (20μm, bar). (C) By immunoblot densitometry, phoS-83 Pxn and total Pxn levels were determined in R3327-5'A cells infected with scramble and shD1. Equal amounts of total protein were loaded in each well. The ratio of phoS83 vs total Pxn was quantified with the Image-Lab 4.0.1 software from BioRad.

SUPPLEMENTARY FIGURE 4

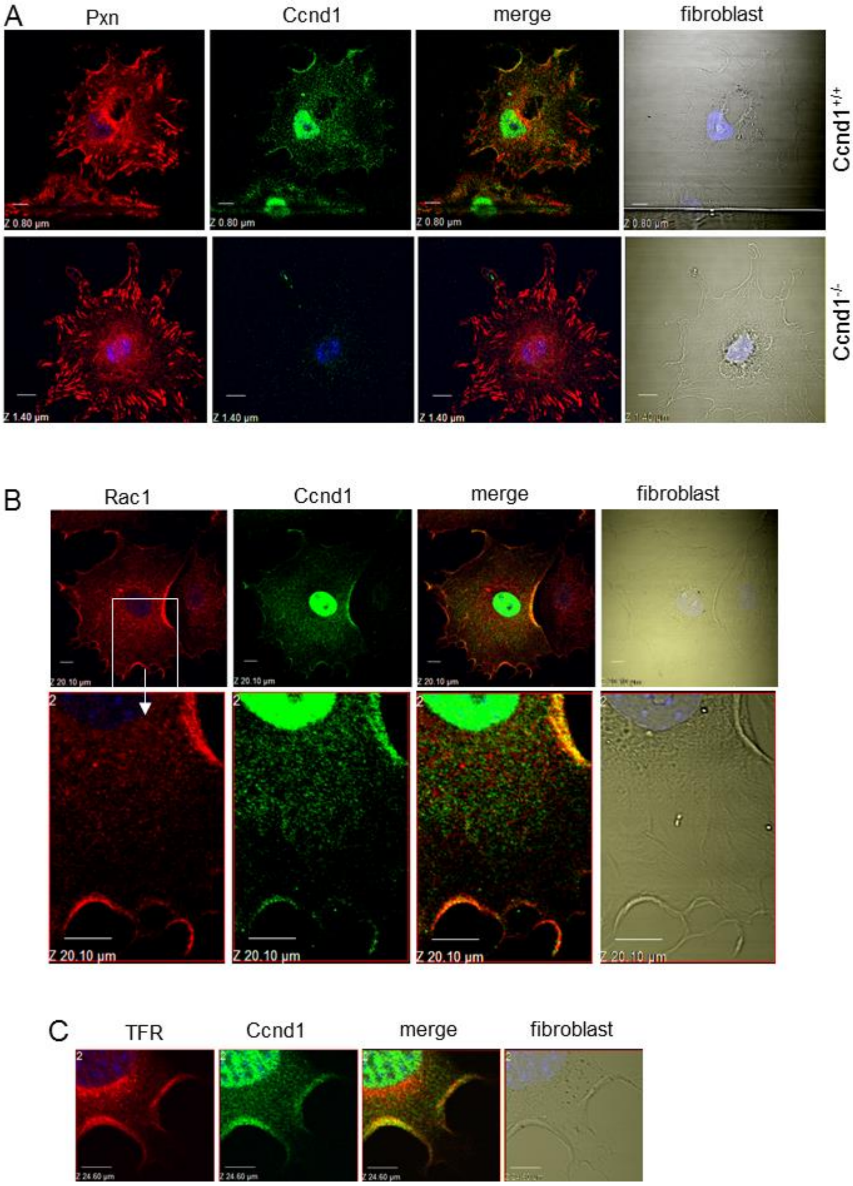

Supplementary Figure 4. Cyclin D1 co-localizes with paxillin in membrane ruffles of fibroblasts. Cyclin D1 co-localizes with paxillin in membrane ruffles. Cells were fixed in 4% paraformaldehyde and permeabilized with 0.2% triton. Images were acquired by confocal microscopy (10μm bar). Nuclei were stained with Hoescht (blue). The antibodies used were: (A) Ccnd1 rabbit monoclonal EP12 (green) and Pxn (mouse monoclonal, red). (B) Rac1 (mouse monoclonal, red). (C) Transferrin receptor (TFR) (mouse monoclonal H68.4, red).

SUPPLEMENTARY FIGURE 5

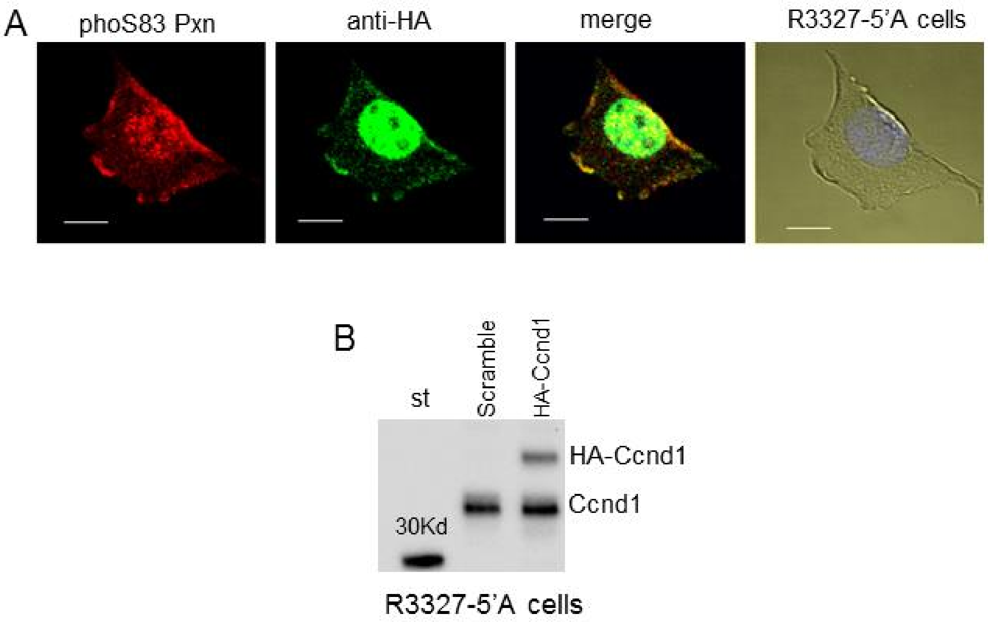

Supplementary Figure 5. Cyclin D1 co-localizes with PhoS83 Pxn along the cell membrane of tumor cells. (A) R3327-5'A cells were infected with scramble and HA-Ccnd1. Cells were fixed in 4% paraformaldehyde and permeabilized with 0.2% triton. Images were acquired by confocal microscopy (10μm bar). Nuclei were stained with Hoescht (blue). The antibodies used were: anti-HA (rat monoclonal 3F10) and anti-Pxn (S83) phospho-specific (rabbit polyclonal). Note that the phospho-specific antibody against Pxn gives a nuclear signal that must be non-specific because total Pxn shows exclusion from the nucleus (referenced in datasheet). (B) By immunoblot total Ccnd1 and infected HA-Ccnd1 levels were determined with monoclonal antibody DCS6 in the R3327-5'A cells used in A. Equal amounts of total protein were loaded in each well.

SUPPLEMENTARY FIGURE 6

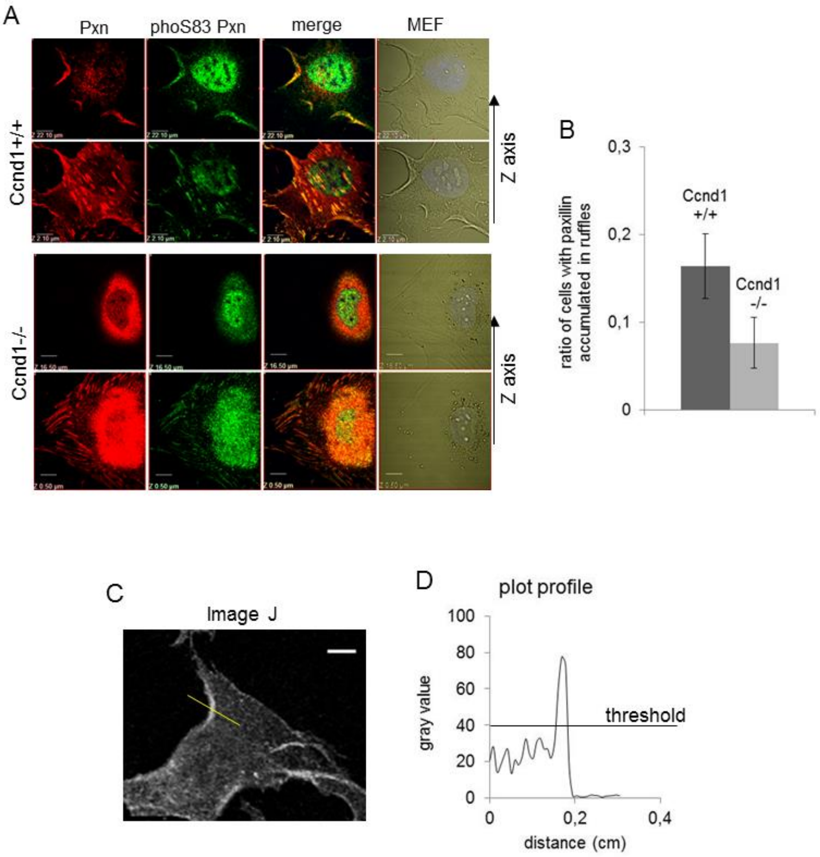

Supplementary Figure 6. Ccnd1$^{-/-}$ primary MEFs show poor localization of paxillin in membrane ruffles. (A) Ccnd1$^{-/-}$ and Ccnd1$^{+/+}$ primary MEFs were fixed in 4% paraformaldehyde and permeabilized with 0.2% triton-X100. Images were acquired by confocal microscopy (10μm bar). Nuclei were stained with Hoescht (blue).The antibodies used were anti-Pxn (monoclonal clone 349, red) and anti-PhoS-83 Pxn (rabbit polyclonal, green) (B) Quantification of cells with Pxn accumulated in membrane ruffles from the images in A. The proportion of cells with Pxn accumulated in ruffles versus total cell number is plotted. The number of cells counted was n≥ 328. Bars indicate the confidence limits for a proportion (α=0.05). (C) Plot profile approach of Image J was used to quantify the accumulation of signal in the membrane (10μm bar). All the images were transformed to eight bits in gray scale values and a threshold above background noise was defined. Cells with at least one region of the membrane with signal levels above the threshold were considered positive, as in D. The yellow line in C indicates the path where the profile is generated. It corresponds to the profile in D.

SUPPLEMENTARY FIGURE 7

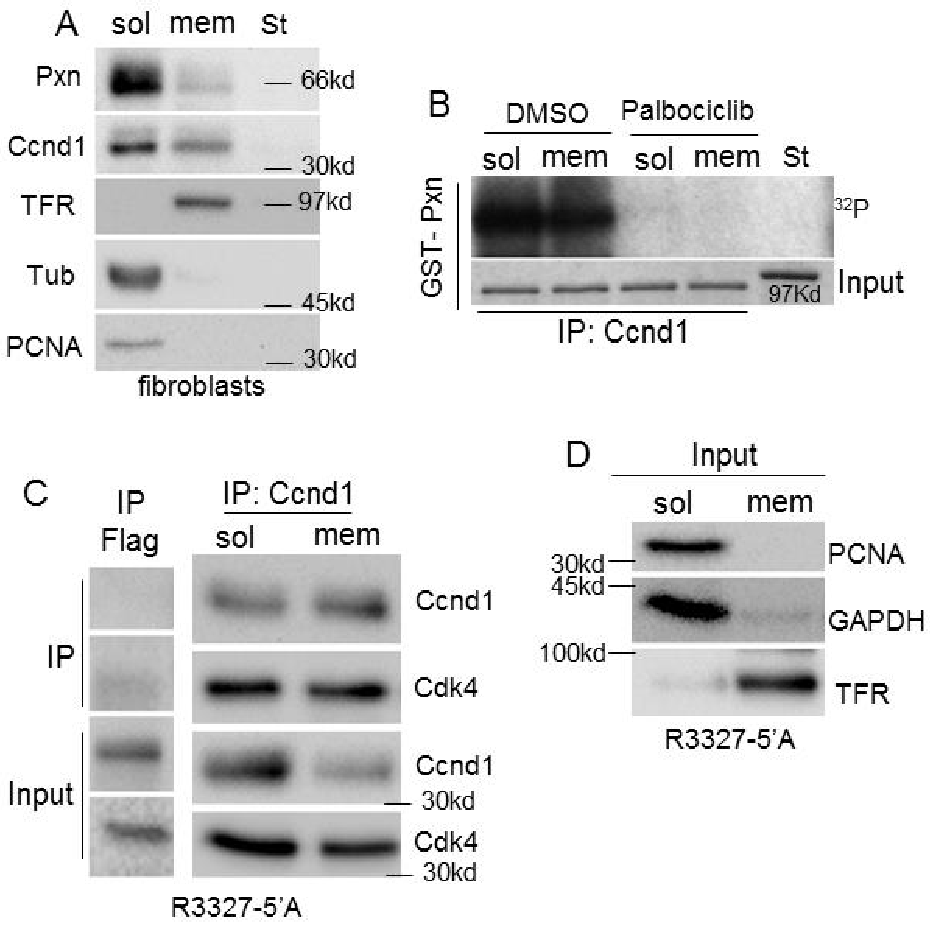

Supplementary Figure 7. Subcellular fractionation of Ccnd1·Cdk4 complexes. (A) Ccnd1$^{+/+}$ Fibroblasts were processed for subcellular fractionation (see "Methods") obtaining soluble (sol) and membrane (mem) fractions. Ccnd1 and Pxn were detected by immunoblot. Tubulin (Tub) as a cytosol marker, Transferrin receptor (TFR) as a membrane marker and PCNA as a nucleoplasm marker were used to control fractionation. (B) Ccnd1 was IP with a rabbit polyclonal antibody from both soluble (sol) and membrane (mem) fractions from rat tumor prostate cells and, the IP samples were used for an "in vitro" kinase assay with GST-Pxn as a substrate. As a control, two reactions were treated with the Cdk4/6 inhibitor Palbociclib at 2 μM. (C) IPs were analyzed by immunoblotting to detect Ccnd1 and Cdk4 in soluble and membrane fractions. As a control a Flag-IP from whole cell extract (wce) was used. (D) GAPDH was used as a cytosol marker.

SUPPLEMENTARY FIGURE 8

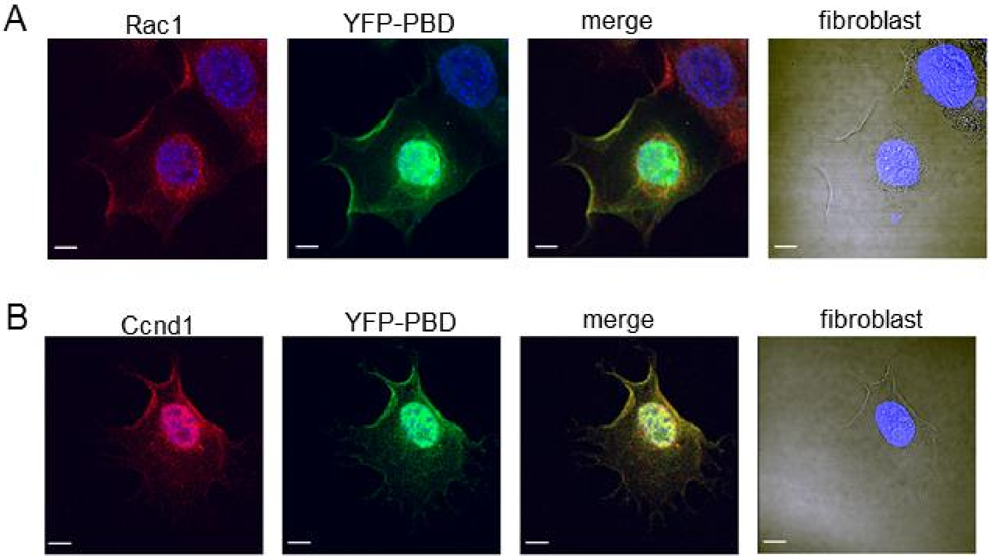

Supplementary Figure 8. Cyclin D1 and Rac1 co-localize with YFP-PBD in membrane. Cells were fixed in 4% paraformaldehyde and permeabilized with 0.2% triton. Images were acquired by confocal microscopy (10μm bar). Nuclei were stained with Hoescht (blue). The antibodies used were: (A) Rac1 (mouse monoclonal, red) and GFP (rabbit Alexa Fluor 488 conjugate, green) (B) Ccnd1 (rabbit monoclonal EP12, red) and GFP (mouse monoclonal 3E6, green).

SUPPLEMENTARY FIGURE 9

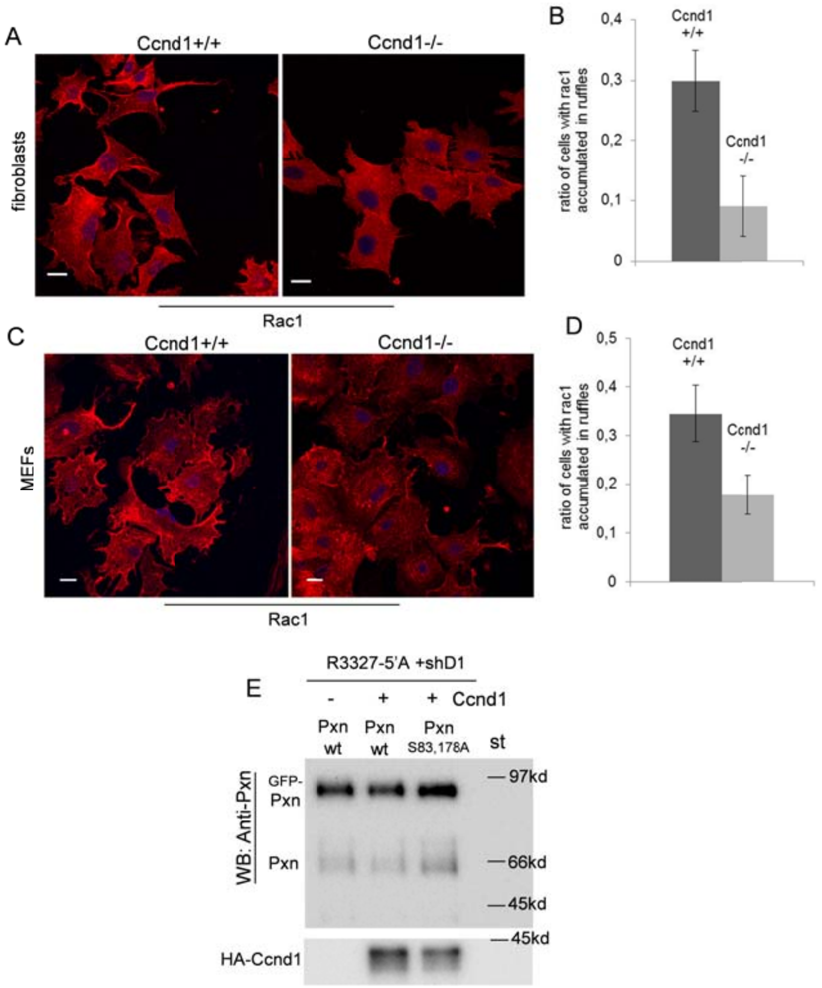

Supplementary Figure 9. Ccnd1$^{-/-}$ primary MEFs show poor localization of Rac1 in membrane. (A and C) Images of *Ccnd1*$^{-/-}$ and *Ccnd1*$^{+/+}$ immortalized and primary MEFs were fixed in 4% paraformaldehyde and permeabilized with 0.2% triton-X100. Images were acquired by confocal microscopy (20μm bar). Nuclei were stained with Hoescht (blue). The antibody used was anti-Rac1 (mouse monoclonal, red) (B and D) Quantification of cells with Rac1 accumulated in membrane from images in A and C. The proportion of cells with Rac1 accumulated in membranes versus total cell number is plotted. The number of cells counted was n≥ 170. Bars indicate the confidence limits for a proportion (α=0.05). (E) Pxn and Ccnd1 levels in the samples corresponding to figure 6D were detected by immunoblot with anti-Pxn (top panel) or anti-HA 3F10 (bottom panel) antibodies.

SUPPLEMENTARY FIGURE 10

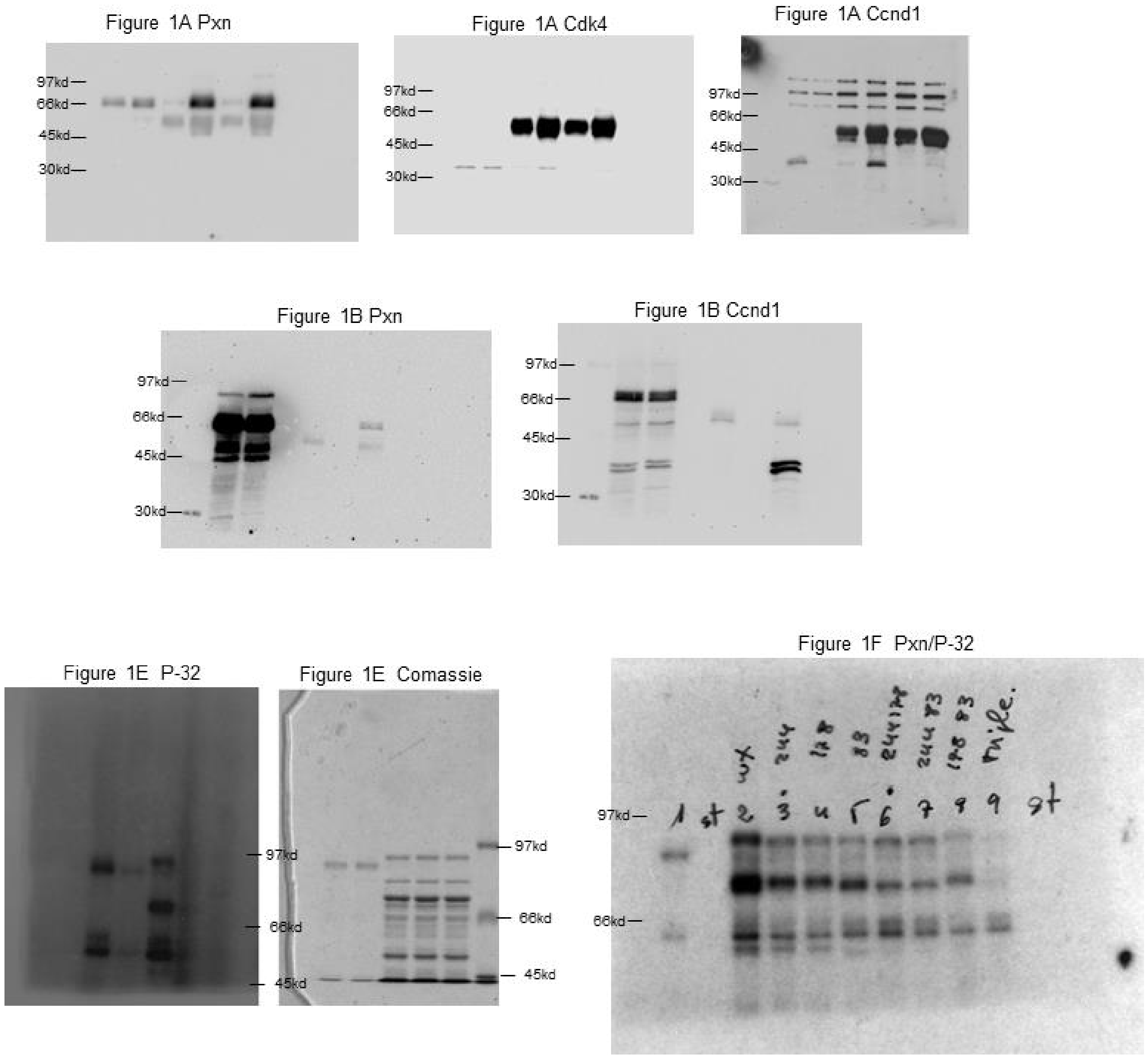

Supplementary Figure 10. Full size scan of western blots shown in Figure 1.

Uncropped blots presented in Figure 1A, 1B,1C and 1F are shown

SUPPLEMENTARY FIGURE 11

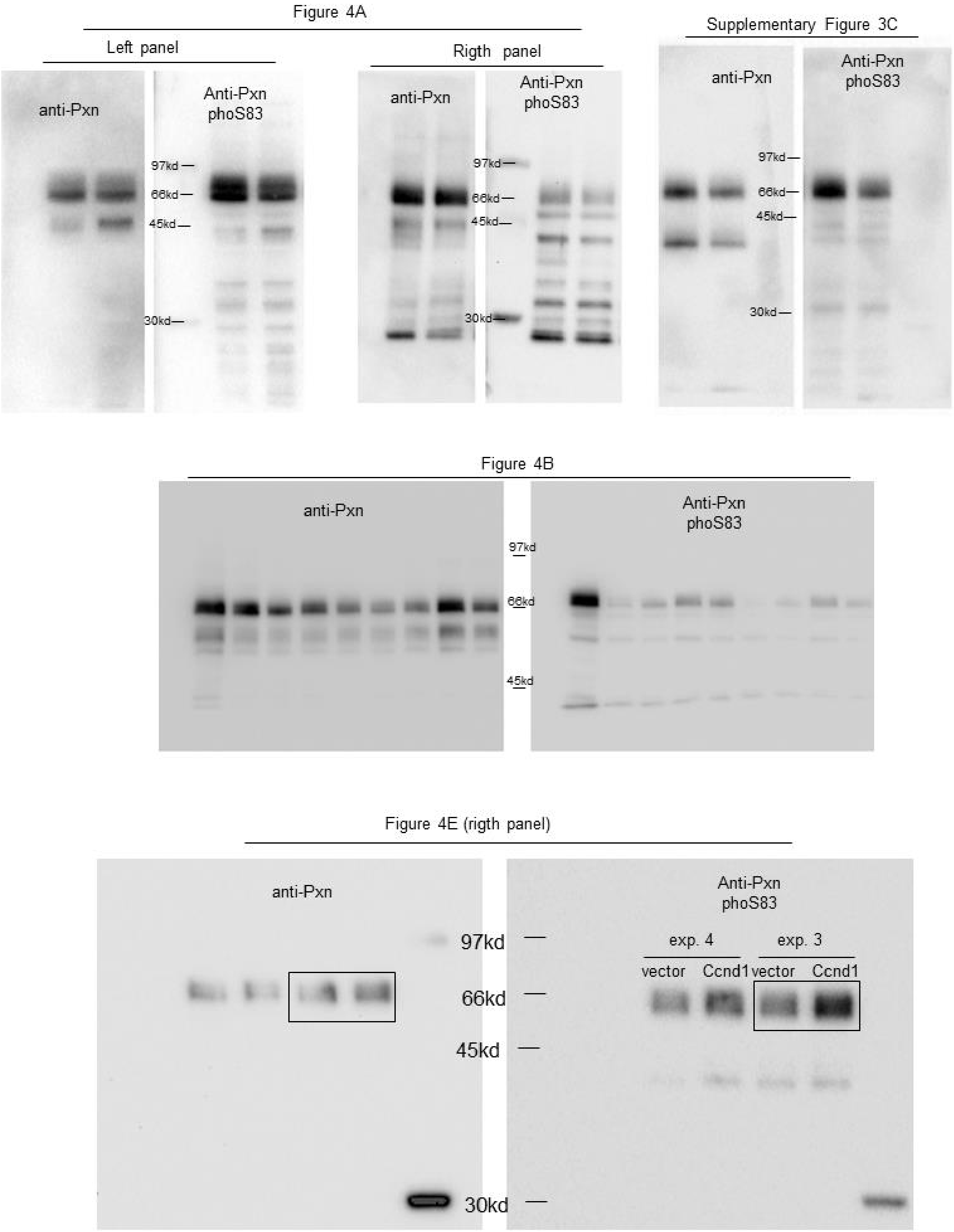

SUPPLEMENTARY FIGURE 11 (cont.)

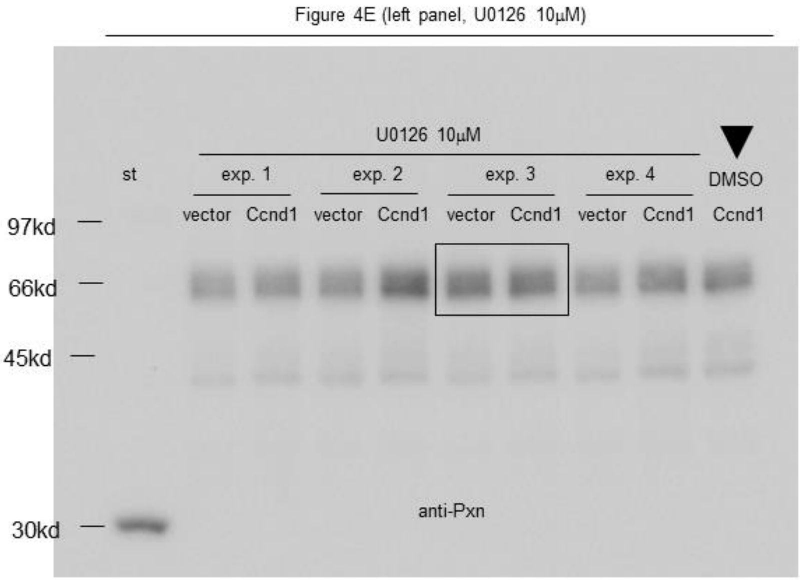

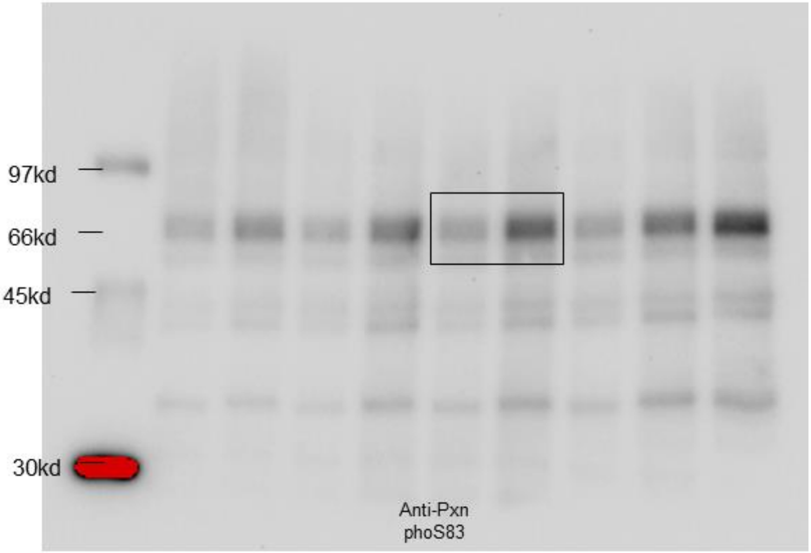

Supplementary Figure 11. Full size scan of western blots shown in Figure 4 and Supplementary Figure 3. Anti-Pxn and PxnphoS83 uncropped blots presented in Figure 4A, 4B, 4E, and in supplementary figure 3C are shown.

SUPPLEMENTARY FIGURE 12

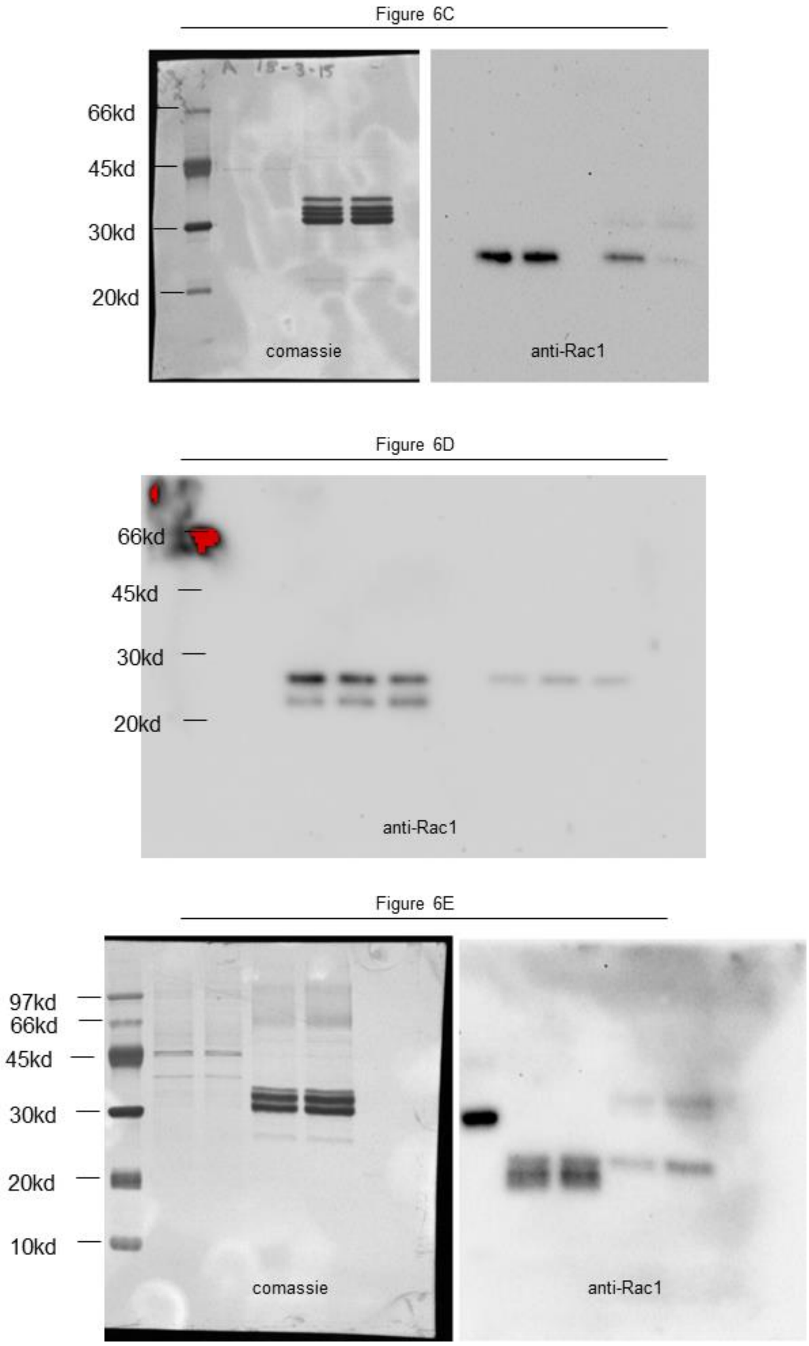

Supplementary Figure 12. Full size scan of western blots shown in Figure 6. Uncropped blots presented in Figure 6C, 6D, and 6E are shown.

SUPPLEMENTARY FIGURE 13

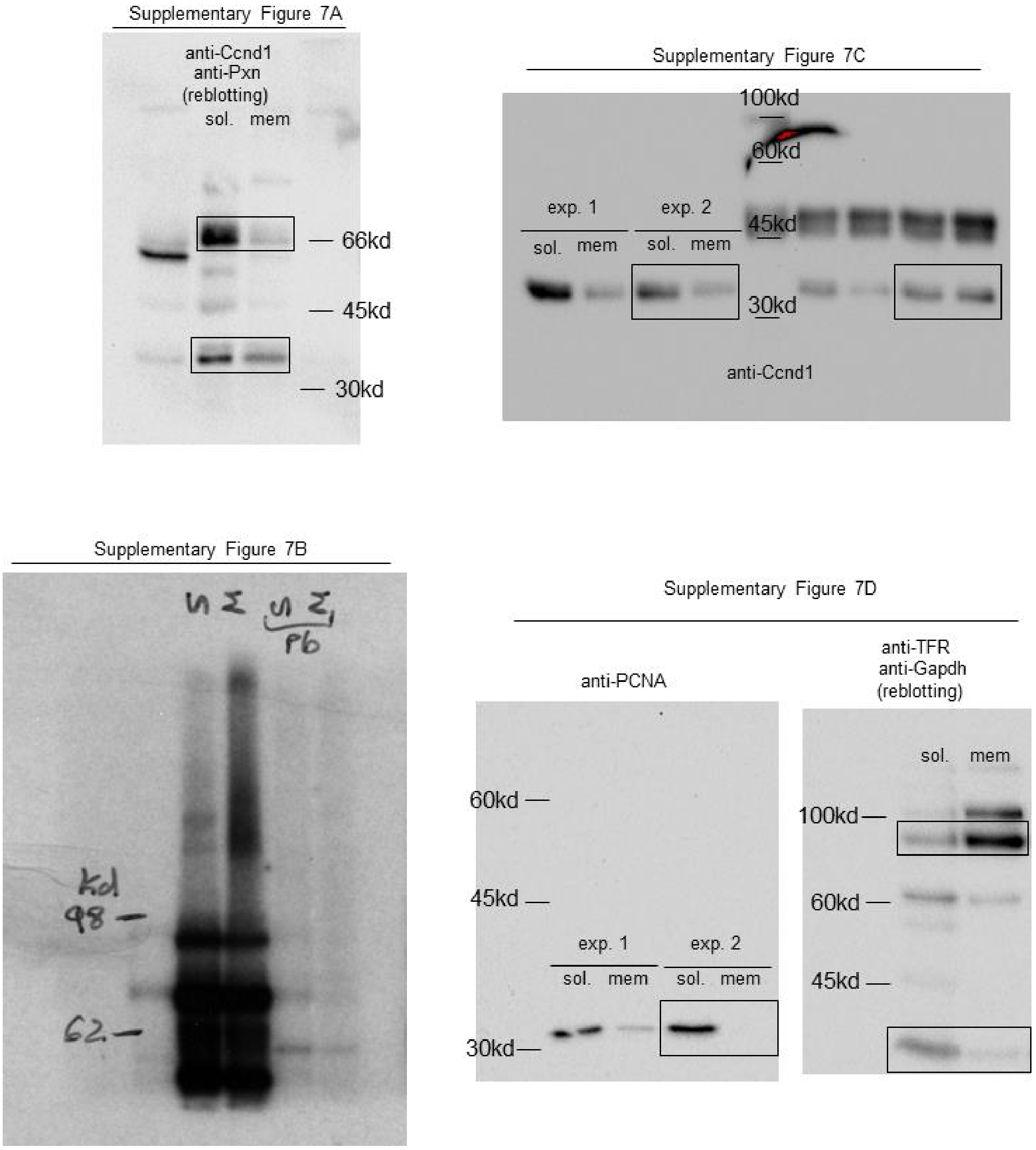

Supplementary Figure 13. Full size scan of western blots shown in Supplementary Figure 7. Uncropped blots for kinase assay, Ccnd1 blot, and fraccionation blots presented in supplementary Figure 7A, 7B, 7C and 7D respectively are shown.

SUPPLEMENTARY TABLE 1

Protein identification by mass spectrometry. Paxillin (Pxn)

samples	Band mobility	1st protein	Score	Coverage (%)	nº peptides	Phospho -peptide
no ATP	1 low	Pxn	1033.78	33.08	18	0
no ATP	2 middle	Pxn	1679.06	47.5	33	0
no ATP	3 high	Pxn	551.05	19.6	11	0
ATP	1 low	No detected				
ATP	2 middle	Pxn	1600.72	42.5	33	0
ATP	3 high	Pxn	730.52	22.8	14	1

SUPPLEMENTARY TABLE 2

Peptide identification by mass spectometry. Peptide sequence corresponds the residues 77 to 88 of mouse Paxillin.

samples	Band mobility	sequence	m/z	z	Δm/z	score	modification
no ATP	1 low	AHQQPPSPLPVY	667.3790	2	45.56	64.25	
no ATP	2 middle	AHQQPPSPLPVY	667.3640	2	23.08	71.51	
no ATP	3 high	AHQQPPSPLPVY	667.4050	2	84.52	47.36	
ATP	1 low						
ATP	2 middle	AHQQPPSPLPVY	667.3700	2	32.08	77.67	
ATP	3 high	AHQQPPSPLPVY	667.3720	2	35.07	44.09	
**ATP***	**3 high**	**AHQQPPSPLPVY**	**707.3420**	**2**	**14.48**	**27.93**	**phospho: 7**
